# Exonization of active mouse L1s: a driver of transcriptome evolution?

**DOI:** 10.1186/1471-2164-8-392

**Published:** 2007-10-26

**Authors:** Tomasz Zemojtel, Tobias Penzkofer, Jörg Schultz, Thomas Dandekar, Richard Badge, Martin Vingron

**Affiliations:** 1 Department of Computational Molecular Biology, Max-Planck-Institute for Molecular Genetics, Ihnestrasse 73, D-14195 Berlin, Germany; 2 Department of Bioinformatics, University of Würzburg, Am Hubland, D-97074 Würzburg, Germany; 3 Department of Genetics, University of Leicester, University Road, Leicester LE1 7RH, UK

## Abstract

**Background:**

Long interspersed nuclear elements (LINE-1s, L1s) have been recently implicated in the regulation of mammalian transcriptomes.

**Results:**

Here, we show that members of the three active mouse L1 subfamilies (A, G_F _and T_F_) contain, in addition to those on their sense strands, conserved functional splice sites on their antisense strands, which trigger multiple exonization events. The latter is particularly intriguing in the light of the strong antisense orientation bias of intronic L1s, implying that the toleration of antisense insertions results in an increased potential for exonization.

**Conclusion:**

In a genome-wide analysis, we have uncovered evidence suggesting that the mobility of the large number of retrotransposition-competent mouse L1s (~2400 potentially active L1s in NCBIm35) has significant potential to shape the mouse transcriptome by continuously generating insertions into transcriptional units.

## Background

LINE-1 elements (L1s) are by far the most abundant class of active autonomous transposons in mammalian genomes [1]. It has been established that active, i.e. retrotransposition-competent, L1 elements in the mouse genome [2,3] outnumber by many fold those found in the human genome [3,4]. This is reflected in more than an order of magnitude difference in the percentage of spontaneous mutations due to L1 activity in mice (~2.5%) compared to that in humans (~0.07%) [5]. As a result, based on recent experimental and bioinformatic data, one might speculate that the high insertional activity of mouse L1s could play a significant part in shaping the structure and expression of the mouse transcriptome [6,7]. Importantly, intronic L1 insertions have been shown to influence the expression of their host genes in a wide variety of ways including retardation of transcriptional elongation [6], transcriptional control [8-10], premature polyadenylation [11], and exon skipping [12].

The process by which L1 sequences inserted within introns are recruited into a mRNA, termed exonization, has been primarily studied by analysis of the human transcriptome [13-16] but to date little evidence has been collected for the mouse [15,17]. In this study, we have assessed the exonization potential of currently active mouse L1 elements. Through detailed analysis of members of active L1 families and cDNA-supported L1 exonization events, we show this potential to be much more significant than previously appreciated. This finding, coupled with the much greater activity of L1s in mouse, suggests that not only have these elements dynamically modified the mouse transcriptome in the past, but continue to do so.

## Results and discussion

### Potentially active LINE-1s in the mouse genome

**Figure 1:**
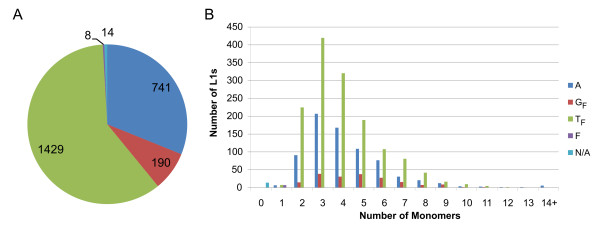
Classification of 2382 potentially active L1 elements residing in the mouse genome sequence (NCBI m35). **A**. Distribution of L1s among subfamilies. A, T_F _and G_F _correspond to active mouse L1 subfamilies. The small number of L1s that appear related to the inactivated F subfamily are marked with F and those lacking monomers are marked with N/A. **B**. Distributions of the lengths of the internal promoter regions among the three active families. The longest promoter discovered is composed of 28 monomers (here included in the 14+ class) and is a feature of a potentially active L1 element belonging to the A subfamily located on chromosome 2 (80663469-80649072).

Using L1Xplorer [3], a suite of automated L1 annotation tools, we identified in the mouse genome sequence (NCBIm35) 2382 potentially active L1 elements (i.e. elements that are full-length and possess intact open reading frames (ORFs) (Fig. 1A, [18]). This contrasts strongly with the 1501 potentially active L1s obtained when analyzing the May 2004 genome release (mm5, build 33) [3] and reflects ongoing finishing of the mouse genome sequence.

Transcription of these open reading frames is driven by the mouse L1 internal promoter, which is built of a variable number of ~200 nt long repeats called monomers. The sequences of active mouse L1s contain related G_F _and T_F _monomers (F-type) as well as unrelated A-type monomers. Consequently, mouse L1s are classified into subfamilies based on the type of the monomer they harbor [2]. Notably we have established, by bioinformatic data mining, that whereas the number of potentially active L1s belonging to the G_F _subfamily agrees with earlier estimates [2], the number of potentially active members of the T_F _and A subfamilies is ~1.6-fold higher than previously (Fig. 1A) estimated. As a result, based on our analysis of genomic sequence data, we conclude that perhaps as many as ~4800 (2*2382) potentially active L1s reside in the diploid mouse genome.

Since L1 activity is expected to correlate with L1 expression level and the latter has been shown to correlate with the length of the internal promoter [19], we aimed to characterize the promoters of the 2382 potentially active L1 elements which we had discovered (Fig. 1B). We found that the G_F _subfamily has the longest average promoter size (~5.5 monomers), followed by the A (~4.4 monomers) and T_F _(~4.1 monomers) families. The annotation of promoter regions is available at [18].

### Splice sites in L1s

**Figure 2:**
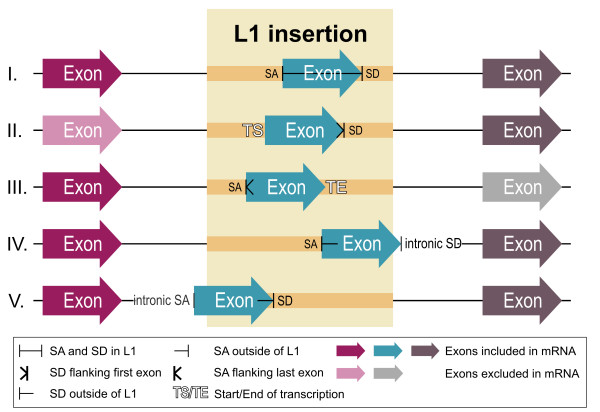
L1 exonization scenarios (I-V) involving sequences belonging to active L1 subfamilies A, T_F_, G_F _and related inactivated F subfamily, as identified in this study. The scenarios I-V are supported by 16, 26, 14, 6, and 2 exonization events, respectively (see Fig. 3 for details of cDNA sequences). SA: splice acceptor, SD: splice donor. In blue: L1-derived exons; in purple and gray: exons of transcriptional units; in light purple and light gray: exons which are not included in transcript due to L1 insertion.

Clearly any dispersed repeat commonly found in intragenic regions has the potential to be exonized due to the fortuitous occurrence of splicing signals in its sequence [20]. Because of their high number (2382 in NCBIm35) and the concomitantly increased potential for insertional activity, it is of particular interest to establish whether the sequences belonging to the currently active mouse L1 subfamilies (T_F_, G_F _and A) contain functional splice sites. We mined cDNA databases (RIKEN and NCBI) to discover putative mouse exonization events involving these sequences (see Materials and Methods). An overview of the different L1 exonization scenarios identified in this study is presented in Fig. 2.

**Figure 3:**
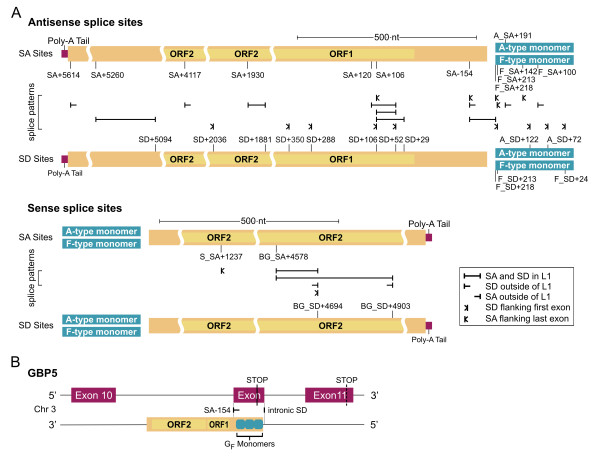
Multiple splice sites are present in antisense and sense L1 sequences (for annotated cDNA examples see [22], for exemplary cDNAs see below). L1Mda2 sequence M13002 was used as a coordinate reference. **A**. Diverse exonization patterns as supported by cDNA evidence. The names of the splice sites incorporate the following information: prefixes of "A_" and "F_" designate sites within A- and F -type (F, T_F_, G_F_) monomers, respectively; SD: splice donor, SA: splice acceptor; the numbering indicates the position of the base after which the cleavage occurs, relative to the start of L1 ORF1, or relative to the start of alignments for monomers (for the alignments see [2, 40]); prefix of "BG_" designates sites found in L1 inserted within an intron of the *beige *gene, prefix of "S" stands for sense splice sites. The blue boxes mark the monomers making up the internal promoter region. Exemplary cDNAs corresponding to the identified splice sites: F_SA+100: *AK017011*, *BC025138*; F_SA+142: *AI194597*, *AK079058*; F_SA+213: *AK081008*; F_SA+218: *AK015559*; A_SA+191: *AK028243*; SA-154: *BC056642*, *AF487898*, *BQ442932*, *AK039191*, *AK043154*, *BG144807*, *AK044020*, *AK145348*, *BB614554*, *BY733866*, *AK076999*, *AK015267*, *AK035725*; SA+106: *AK080034*, *AY167972*, *BG144807*, *BY733866*, *AK015267*, *AK007310*; SA+120: *AK006905*, SA+1930: *NM_177142*; SA+4117: *AK034994*; SA+5260: *AF529222*; SA+5614: *AK032656*; F_SD+24: *AK035725*; F_SD+213: *AK081008*; F_SD+218: *AK035725*; A_SD+72: *AK077067*, *AK015711*; A_SD+122: *AK032374*, *BC017615*, *AK015277*, *AK006354*; SD+29: *BY733866*; SD+52: *AK080034*, *AY167972*, *BG144807*, *AK006905*, *AK007235*, *AK161293*, *AK132928*, *AK135585*, *BB614554*, *AK016072*, *AK015559*, *AK076999*, *AK015267*, *AK015548*, *AK015778*, *AK015845*; SD+106: *AK015524*; SD+288: *AK076828*, *AK006905*, *AK015267*; SD+350: *AK017011*; SD+1881: *NM_177142*; SD+2036: *NM_177142*; SD+5094: *AF529222*; BG_SA+4578: insertion in *beige *gene (for sequence see the online annotation); S_SA+1237: *AK040102*; BG_SD+4903: *AK031201*, *AK032656*; BG_SD+4694: *AK134759*, *AK038418*, *DV059289*, *AK015958*, *AK034994*, insertion in beige gene. **B**. Insertion of L1 G_F _element in the intron of *GBP-5 *gene introduced a SA site (SA-154) and resulted in creation of a novel exon coding for the C-terminal and bearing a new stop codon (solid vertical line) (cDNA transcripts GBP-5a, b: gi: *24266664*, *26326418*).

Fig. 3A contains a summary of antisense (upper) and sense (lower) splice sites in L1 sequences, as identified by analysis of cDNA sequences. All of these splice donor sites (SD) and splice acceptor sites (SA) are consistent with the classical GT/AG splice junction motifs [21]. For details of 52 fully annotated examples see: [22].

Of the 52 discovered events 43 involved the exonization of antisense L1 sequences. Similarly, the authors of a very recent study investigated exonization of transposable elements and reported the greater number of the antisense orientation L1 exonization events [23]. The observed greater number of L1 exonizations in the antisense may result from the antisense orientation bias of intronic L1s (see **Antisense splice sites vs. antisense L1 insertional bias **and **Conclusion**).

The most frequently used acceptor and donor splice sites we have identified are SA-154 (located in the antisense strand of 5' UTR) and SD+52 (in the antisense strand of ORF1), supported by 13 and 16 different cDNA transcripts, respectively (Fig. 3A).

A classic example of sense orientation L1 exonization was previously reported when the insertion of a ~1100 bp 3' fragment of a L1 T_F _element within an intron of the *beige *gene caused a disease-specific mutation in mouse [17]. Usage of the two SD sites, BG_SD+4694 and BG_SD+4903, identified in the latter study, was also evident in 6 and 3 different cDNAs, respectively, in our data set (see Fig. 3A and online annotation at [22]).

### Diverse lengths of exonized L1s

Clearly, truncated and rearranged L1s provide different splice sites. The shortest exonized L1 insertion we have annotated is only 164 bp long (see online annotation of AK032656) and provides the antisense SA+5614 site located at the polyadenylation signal (notably, the polypyrimidine tract here is derived from the polyadenylation signal of L1). As shown in this study (see [22]) the diverse range of lengths of exonized L1 insertions renders numerous possibilities for the combinatorial usage of the splice sites. Conversely, as evidenced by cDNA AK034994, two separate antisense (237 bp) and sense (1117 bp) L1 insertions cooperatively provide SD and SA sites to create a L1-derived exon.

### Evidence for an antisense promoter in mouse L1s

**Figure 4:**
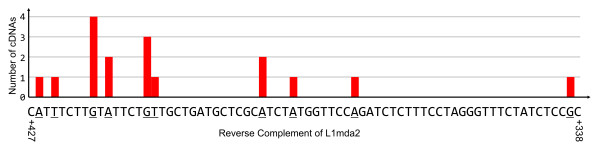
Distribution of transcriptional start sites within a region of antisense sequence of the ORF1. The coordinates are with respect to the start of the ORF1 in L1mda2 sequence (M13002). The Y axis represents the number of cDNAs supporting each transcriptional start site location. In total, 17 cDNAs support the TSs in this region: AK017011, AK076828, AK006905, AK007235, AK015524, AK161293, AK132928, AK135585, AK077067, AK016072, AK015559, AK076999, AK015267, AK015548, AK015778, AK015845, AK015266.

As shown in Fig. 3A (scenario II in Fig. 2), we observed an exonization scenario in which first exons are created from intronic antisense L1 sequences (see [22] for examples). In these cases, first exons are spliced to the downstream exons via SD sites present within antisense L1 sequences. This might support the existence of regulatory motifs within mouse antisense L1 sequences that can initiate transcription, as has been reported for the antisense promoter in human L1s [8-10,24]. We identified a region of transcriptional start site within the antisense sequence of ORF1, which is supported by 17 different cDNAs (Fig. 4). This region could be the site of a novel mouse L1 antisense promoter, but experimental studies would be required to confirm this. Transcription of L1-derived first exons, that subsequently capture downstream exons which encode intact protein domains, may constitute a mechanism for the generation of novel functional coding transcripts through "gene breaking", as has been described in humans [24]. For example, in one of our annotated cDNA examples, AK006905, the SD+288 site present in ~6 kbp L1 T_F _insertion is used to link L1-derived sequence with downstream exons encoding the C-terminus of DNA directed polymerase iota (gi: 6755273). This mRNA transcript, would encode a 254 amino acid (a.a.) protein containing two ubiquitin-binding motifs (UBM) [25]. Also in this example, two more splice sites originating from the same ~6 kbp L1 T_F_. insertion, SA+120 and SD+52 generate an L1-derived exon. A similar exonization pattern, leading to first exon generation, is also evident in the transcript AK015267 where another ~6 kbp L1G_F _insertion provides SD+288, SA+106 and SD+52 sites, as well as SA-154. In the latter case SA+120 is substituted for the SA+106 site located nearby (see online annotation).

### Coding potential of antisense L1-derived exons

Provocatively it has been proposed that, in general, repeat exonization via alternative splicing may constitute a vehicle for the exaption of repeat sequences into novel functions [20,26,27]. In line with this, a recent experimental study demonstrated that arbitrary sequences can evolve towards functionality when fused with other pre-existing protein modules [28].

Our analyses of cDNAs have revealed that exonized antisense L1 sequences have the potential to code for parts of ORFs. For example, the alternative transcripts of the GBP-5 gene (gi: 24266664, 26326418) contain an L1-derived exon which contains sequences from three G_F _monomers. The antisense sequences of each of the three monomers can be translated into peptides which are ~60 a.a. in length yielding a novel 174 a.a. long C-terminus of *GBP-5 *protein (see Fig. 3B and also [22]). Although, clearly, the resulting protein variant is mouse-specific, it was noted that the alternative C-terminus variant of GBP-5 exists in humans (AF328727) and that both mouse and human variants lack the C-terminal CaaX isoprenylation motif [29,30], which might be of physiological importance.

### Exonization potential of active L1s

**Figure 5:**
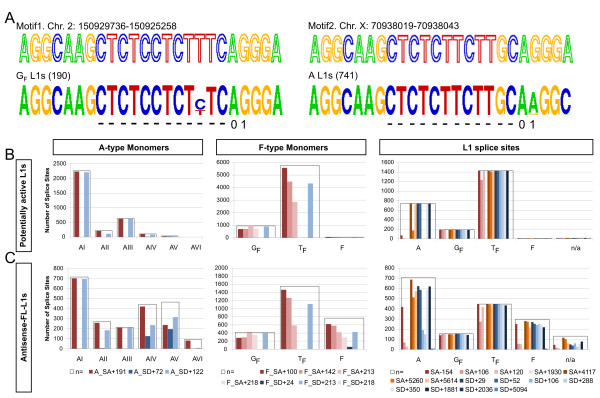
Annotation of antisense splice sites in different subfamilies of putatively active L1s and antisense intronic FL L1 insertions. **A**. For illustration the polypyrimidine tracts for the site SA-154 are shown. Here, the splice donor AG motif is present only in a small fraction of full-length intact elements belonging to the A subfamily (741), whereas it is intact in G_F _subfamily (190). "01" marks the location of AG splice acceptor motif; "-" designates the position of the polypyrimidine tract. Exemplary Motif1 and Motif2 sequences, containing the functional SA-154 splice site, are evidenced by mapping of cDNAs (AK145348, BG144807, respectively) to the corresponding genomic locations containing L1s (NCBIm35). **B**. Conservation of antisense GT/AG splice motifs in potentially active L1s. **C**. Conservation of antisense GT/AG splice motifs in antisense intronic FL L1 insertions. "n = " indicates the number of annotated L1s/monomers. Legend: cDNA-identified splice sites.

To examine the exonization potential of active L1 elements, we analyzed the residues at the positions corresponding to the cDNA-identified splice sites in a set of 2382 sequences of potentially active L1s. For this, we utilized the L1Xplorer annotation pipeline and created a set of customized modules (see Materials and Methods for details). Since all of the identified splice sites were classical GT-AG sites, we defined the presence of these nucleotides as a criterion for a functional splice site. In a total of 2382 full-length intact LINE-1 elements 45471 donor and 47848 acceptor splice sites were annotated (see Fig. 5B for summary on conservation of antisense splice sites, Additional File 1, for details on conservation of sense splice sites and for splice site annotation in L1 sequences see [18]).

We rendered sequence logos of the sequences corresponding to the identified acceptor and donor motifs in L1s (Additional File 2). In analyzing logos of the acceptor sites we observed polypyrimidine tracts which are typical of consensus splice sites in mouse genes [31] (see Fig. 5A). The presence of these motifs supports the putative functionality of annotated acceptor sites in active L1s.

As illustrated for the case of SA-154 site in Fig. 5A, family-specific patterns of splice motif conservations are observed in potentially active L1 sequences. More cases, such as that of SA+106, SD+288 sites that are AG/GT intact only in F-type mouse L1s, are highlighted in Fig. 5B.

We also found three splice sites SD+5094, SD+2036 and SA+1930 that are not intact in any subfamily of the potentially active L1 sequences and have been generated by single nucleotide mutations leading to functional "GT" and "AG" motifs (GG->GT, CT->GT and AT->AG respectively, see Additional File 2). The SA+1930 acceptor splice site contains the 11 bp-long polypyrimidine tract which is present in all sequences of potentially active L1s (Additional File 2). Thus the single nucleotide T->G mutation could activate this cryptic acceptor splice site in potentially active L1 sequences.

### Splice sites in intronic full-length L1 sequences

The amount of L1 sequence residing in mouse introns is ~25% of the total genomic L1 content and as much as 8% of all intronic sequences are L1-derived nucleotides (based on our cumulative analysis of annotated transcriptional units found in Refseq, Known Genes and Ensembl from the UCSC mm7 Dataset). This sequence is comprised mostly of truncated L1 sequences: according to RepeatMasker annotation of NCBIm35 (UCSC mm7) more than 92% of intronic L1 sequences are less than 1000 nt long. As shown above, even these short sequences can be subject to exonization. It is expected, however, that intronic full-length (FL) L1 insertions have a much higher exonization potential since they contain multiple splice sites and, for example, only FL L1s will include 5' promoter regions containing splice sites.

We identified 1739 antisense and 1014 sense intronic FL (greater than 5 kbp in length) L1 insertions. As revealed by our L1Xplorer annotation, these belong in large part (75% in sense and 78% in antisense) to the active A, G_F _and T_F _families but some (17% in sense and 16% in antisense) belong to the inactivated F subfamily (Additional File 3). Similar to potentially active L1s, splice sites are largely intact in intronic FL L1 insertions (Fig. 5C, Additional File 1, Additional File 2). Multiple cDNAs confirm exonization of antisense sequences of FL elements (see [22]). In particular, antisense sequences of two intronic potentially active L1 T_F _elements are exonized via the SD+52 site to create first exons in cDNAs AK132928 and AK007235.

By analysing three groups of gene annotations (cDNA and corresponding DNA), we identified as many as 1259 Ensembl Genes, 1436 UCSC Known Genes and 858 RefSeq Genes with at least one FL antisense intronic L1 insertion and 718 Ensembl Genes, 797 UCSC Known Genes and 464 RefSeq Genes that contained at least one intronic FL sense L1 insertion. Hence, the prediction of potential splice sites within FL intronic L1s may be an important consideration for researchers studying transcripts of particular genes. We have added the annotation of intronic FL L1 elements to L1Base, which is available at [18].

### Antisense splice sites vs. antisense L1 insertional bias

The existence of antisense splice sites is highly intriguing, particularly in the light of ~2 fold antisense orientation bias of L1s located in introns of transcriptional units [32]. This orientation bias is especially evident when comparing regions immediately flanking transcriptional start sites (TSS) and transcriptional end sites (TES) (Additional File 4).

However, this global picture, which is based on all L1 insertions, does not provide information on whether the bias results from processes acting on a long time scales or rather is already reflected in young L1 insertions. To gain insight into this issue we specifically looked at young intronic FL L1 insertions.

The ratio of antisense to sense FL intronic insertions is ~1.7 and chi-square testing established that this ratio is significantly different from random insertion orientation model (chi^2^: p < 0.0001), where either orientation is equally likely.

We set out to investigate, if this insertional bias is still evident among younger intronic insertions. For this analysis we utilized the set of the potentially active L1s (i.e. full-length elements with intact ORFs) that were inserted within introns. This is more stringent than analysing FL insertions with disrupted ORFs, since elements with intact ORFs are likely to be younger due to the L1 proteins' *cis*-preference towards their encoding RNA [33]. ~28% (657) of putatively active sequences reside in introns (393 in antisense, 270 in sense, 6 both in sense and antisense, ratio ~1.46). The chi-square test indicated that this is again highly significantly different from a random insertion model (chi^2^: p < 0.0001). This result suggests that if insertion orientation is random for *de novo *insertions, and the observed bias towards antisense insertion occurs due to selection against sense insertions, this selection process is rapid.

The family distribution for the antisense intronic L1 insertions (Additional File 3A) is very similar to the FL L1s in intergenic regions (Additional File 3B). This is what one would expect to see under the assumption that the sequences of all L1 families equally impact the genes they insert into (i.e. there is no negative selection against any particular subfamily). However, we did observe a difference in the distribution of T_F _and A subfamilies between intronic sense FL insertions (Additional File 3B) and intragenic FL insertions (chi^2^: p < 0.0001). One might argue here that negative selection appears to have acted specifically on the intronic sense L1 insertions belonging to the A subfamily, but it is not clear why this might be.

## Conclusion

We have shown that active mouse L1 elements contain functional splice sites within their antisense sequences using evidence from exonization events in mouse cDNA libraries. This is especially interesting in the light of the antisense insertional bias of *de novo *L1 insertions. A recent experimental study addressed the molecular nature of this phenomenon by showing that sense insertions of mouse L1 T_F _element reduce transcript levels and impair their structure. By contrast antisense intronic insertions have little or no effect on transcript elongation and abundance [32]. These data suggest that the apparently benign nature of antisense intronic insertions and the presence of functional antisense splice sites can lead to the frequent exonization of L1 sequences, as we have observed in our dataset. Further, the conservation of these splices sites in L1 families known to be currently active in the mouse genome strongly implies that generation of L1-exonized transcripts is ongoing, and thus represents a driver of transcriptome evolution. However, it has to be said that this picture is complicated by many factors, particularly since the combination of intronic environment, the size and exact structure of the L1 insertion can impact upon its exonization potential, resulting in gene and insertion specific patterns of exonization. With this caveat, the evidence for inclusion of L1 sequences in transcripts and the high activity of the many LINE-1s in the mouse genome, suggests that their integration into introns has significant ongoing potential to shape the structure of the transcriptome, and ultimately, the proteome [26], in the course of evolution.

## Methods

### Identification of splice sites

We screened mouse cDNA databases (FANTOM3 [34] and NCBI) with RepeatMasker to identify cDNAs containing L1 sequences. The splice sites within L1 sequences were identified using a combination of the following tools. We used BLAT [35] to identify the genomic localization of the cDNAs on the mouse genome (NCBIm35). The genomic regions were extracted either from ENSEMBL [36] or the NCBI Nucleotide Database [37]. SPLIGN [38] was used to split the cDNAs into exons. RepeatMasker was used to identify the repeats in genomic regions corresponding to the mapped cDNAs. Family classifications of L1s were carried out with RepeatMasker and a customized version of the monomer search module of L1Xplorer, which uses Matcher from the EMBOSS package [39] and template sequences for A- and F-type monomers [2,40]. This allowed us to specifically identify the candidate splice sites that occur in sequences belonging to active L1 families. Because of their sequence similarity to the active G_F _and T_F _subfamilies, we also included in our analysis cases of exonization of sequences from the inactivated F subfamily. To expedite the splice site annotation process we developed, using PHP [41] and MySQL [42], a web interface which allowed for manual data curation. Furthermore a set of perl scripts has been developed to interact with the data and compute statistics. The online database containing annotations of cDNA transcripts with exonized L1 sequences [22] is a read-only version of the annotation system.

### Annotation of Splice Sites in potentially active L1s and intronic FL L1s

We used a set of potentially active L1s, as identified in NCBIm35 [18], to examine the potential location of splice sites. In order to compile a set of FL intronic L1 insertions we extracted L1s residing in introns and spanning more than 5000 nt using the RepeatMasker annotation present in Ensembl (Mus musculus v38.35 [36]). The data was then split into sense and antisense L1s. To automatically annotate the presence or absence of the splice sites two L1Xplorer modules were developed: the first module utilizes the alignment-based search as determined by Matcher [39] for splice sites within monomers and the second utilizes a HMMer-based search [43] for splice sites within remaining parts of L1s.

## Authors' contributions

TZ and TP designed the study and performed the analysis, drafted and wrote the manuscript. JS and TD participated in writing the manuscript. RB participated in the design of the study and wrote the manuscript. MV participated in the design of the study and wrote the manuscript. All authors read and approved the final manuscript.

## Supplementary Material

Additional File 1 Annotation of sense splice sites in different subfamilies of potentially active L1s and sense intronic FL insertions. Conservation of AT/GT splice motifs. "n = " indicates the number of annotated L1s. Click here for file

Additional File 2 Logos of annotated splice sites motifs in sequences of potentially active L1s and FL intronic L1 insertions. Motif: sequences of functional splice sites identified via mapping of cDNAs to the mouse genome (see Fig. 3 and [22] for cDNA sequences). Click here for file

Additional File 3 Distribution of antisense (1739) and sense (1014) intronic full length L1s (A) and full length intergenic L1s (10671) (B) among subfamilies. See online annotation at [18]. Click here for file

Additional File 4 Distribution of Line-1 elements around the transcriptional start sites (TSS) and transcriptional end sites (TES) of ~80000 transcriptional units (combined Ensembl, Refseq, UCSC mm7 annotations). **A**. L1 insertions found at +/-50 kbp from TSS and TES of transcriptional units. **B**. Intronic-only L1 insertions. X-axis of the left-hand chart in A and B: distance from TSS, X-axis of the right-hand chart in A and B: the distance from TES. The primary (leftmost) Y-axis shows the number of nucleotides (base pairs) of L1 sequence in sense (red) and antisense (yellow) orientation. The secondary (rightmost) Y-axis: shows the ratio of antisense to sense insertions (black line). Data are plotted in bins of 100 bp. Web data: Annotation of 52 exemplary cDNAs is available at [22]. The database containing the sequences and annotations of potentially active L1s and full length (FL) intronic L1s is available at [18]. Click here for file
