# Supplementary figures and images for: Exonization of active mouse L1s: a driver of transcriptome evolution?
Source: BMC Genomics. 2007 Oct 26;8:392. doi: 10.1186/1471-2164-8-392 (PMC2176070; doi:10.1186/1471-2164-8-392)

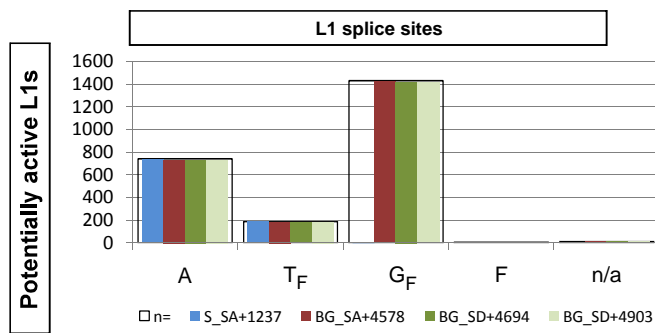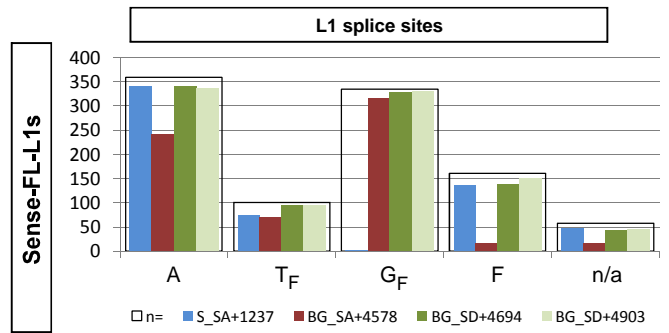

Supplement: Additional File 1 — Annotation of sense splice sites in different subfamilies of potentially active L1s and sense intronic FL insertions. Conservation of AT/GT splice motifs. "n = " indicates the number of annotated L1s. [file 1471-2164-8-392-S1.pdf]

A

Antisense intronic FL L1s

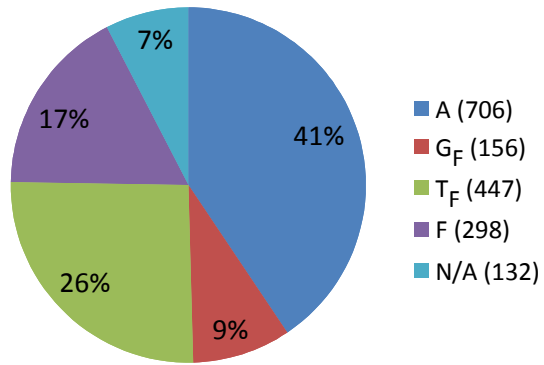

Sense intronic FL L1s

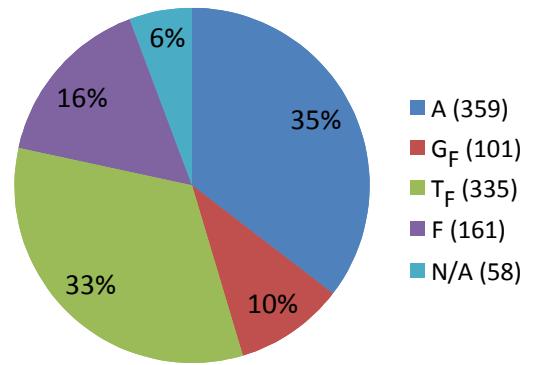

B

FL intergenic

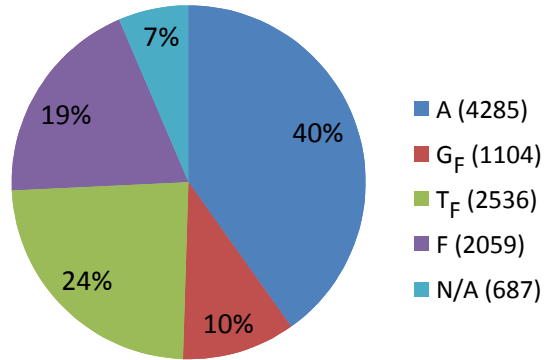

Supplement: Additional File 3 — Distribution of antisense (1739) and sense (1014) intronic full length L1s (A) and full length intergenic L1s (10671) (B) among subfamilies. See online annotation at [18]. [file 1471-2164-8-392-S3.pdf]

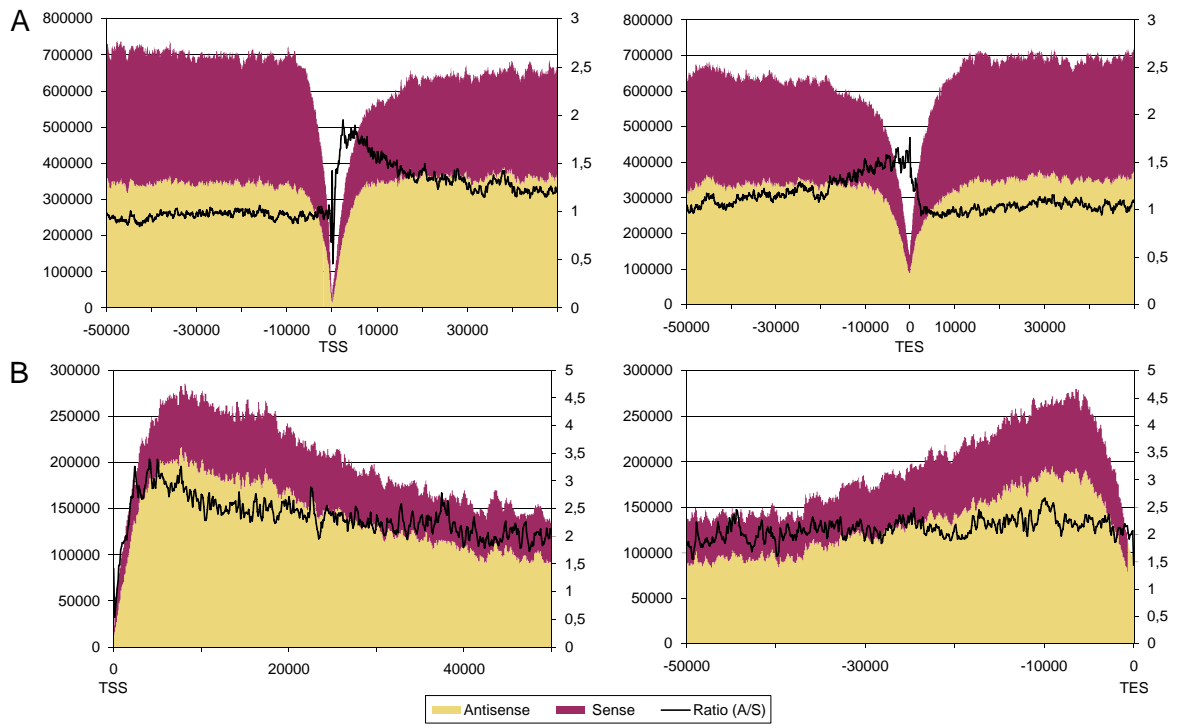

Supplement: Additional File 4 — Distribution of Line-1 elements around the transcriptional start sites (TSS) and transcriptional end sites (TES) of ~80000 transcriptional units (combined Ensembl, Refseq, UCSC mm7 annotations). A. L1 insertions found at +/-50 kbp from TSS and TES of transcriptional units. B. Intronic-only L1 insertions. X-axis of the left-hand chart in A and B: distance from TSS, X-axis of the right-hand chart in A and B: the distance from TES. The primary (leftmost) Y-axis shows the number of nucleotides (base pairs) of L1 sequence in sense (red) and antisense (yellow) orientation. The secondary (rightmost) Y-axis: shows the ratio of antisense to sense insertions (black line). Data are plotted in bins of 100 bp. Web data: Annotation of 52 exemplary cDNAs is available at [22]. The database containing the sequences and annotations of potentially active L1s and full length (FL) intronic L1s is available at [18]. [file 1471-2164-8-392-S4.pdf]
