# Supplementary material for: Exonization of active mouse L1s: a driver of transcriptome evolution?
Source: BMC Genomics. 2007 Oct 26;8:392. doi: 10.1186/1471-2164-8-392 (PMC2176070; doi:10.1186/1471-2164-8-392)
Supplement: Additional File 2 — Logos of annotated splice sites motifs in sequences of potentially active L1s and FL intronic L1 insertions. Motif: sequences of functional splice sites identified via mapping of cDNAs to the mouse genome (see Fig. 3 and [22] for cDNA sequences). [file 1471-2164-8-392-S2.pdf]

SA-154

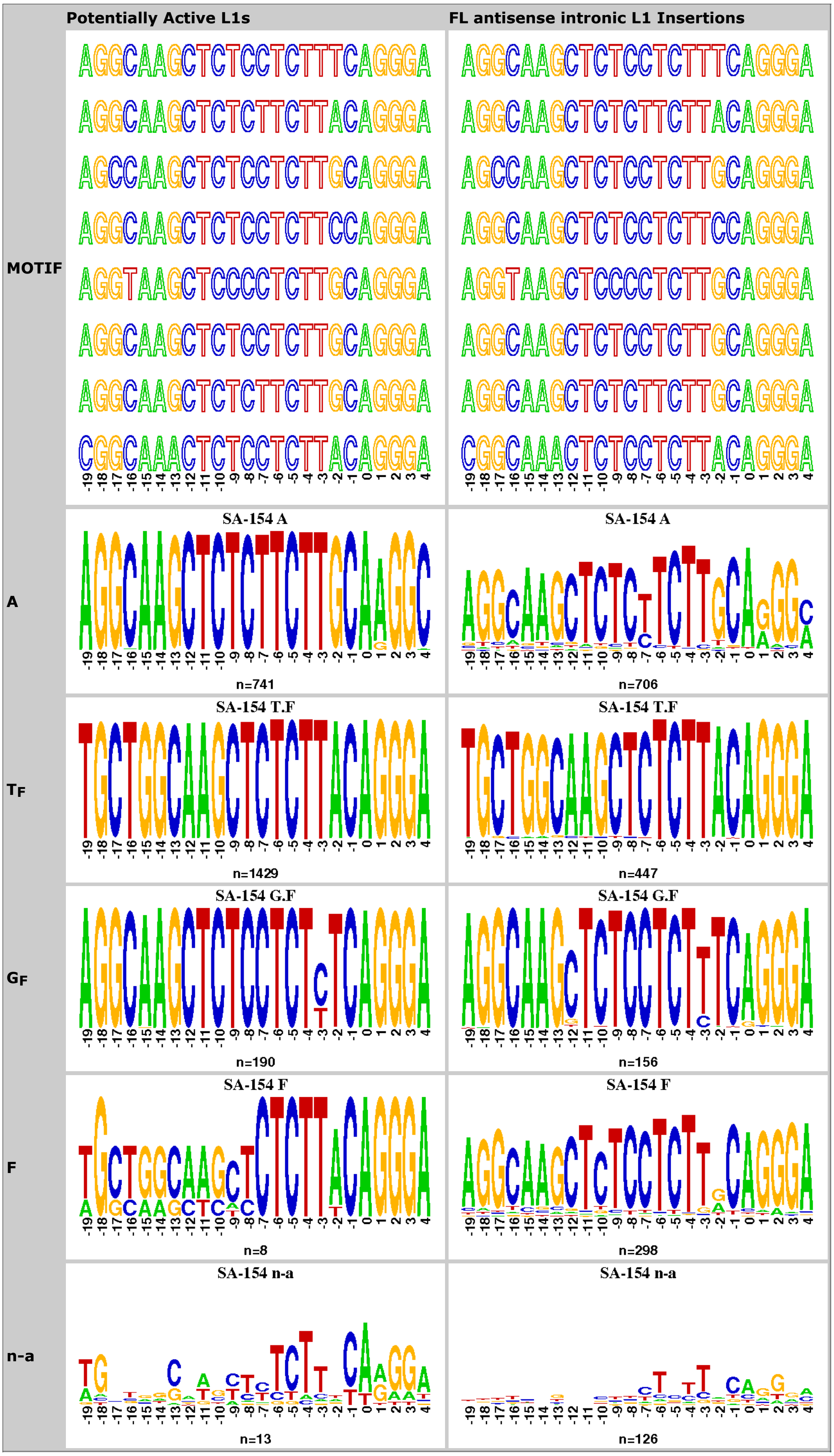
Potentially Active L1s
FL antisense intronic L1 Insertions
MOTIF
AGGCAAGCTCTCCTCTTTCAGGGA
AGGCAAGCTCTCTTCTTACAGGGA
AGCCAAGCTCTCCTCTTGCAGGGA
AGGCAAGCTCTCCTCTTCCAGGGA
AGGTAAGCTCCCCTCTTGCAGGGA
AGGCAAGCTCTCCTCTTGCAGGGA
AGGCAAGCTCTCTTCTTGCAGGGA
CGGCAAACTCTCCTCTTACAGGGA
A
SA-154 A
n=741
n=706
TF
SA-154 T.F
n=1429
n=447
GF
SA-154 G.F
n=190
n=156
F
SA-154 F
n=8
n=298
n-a
SA-154 n-a
n=13
n=126

SA+106

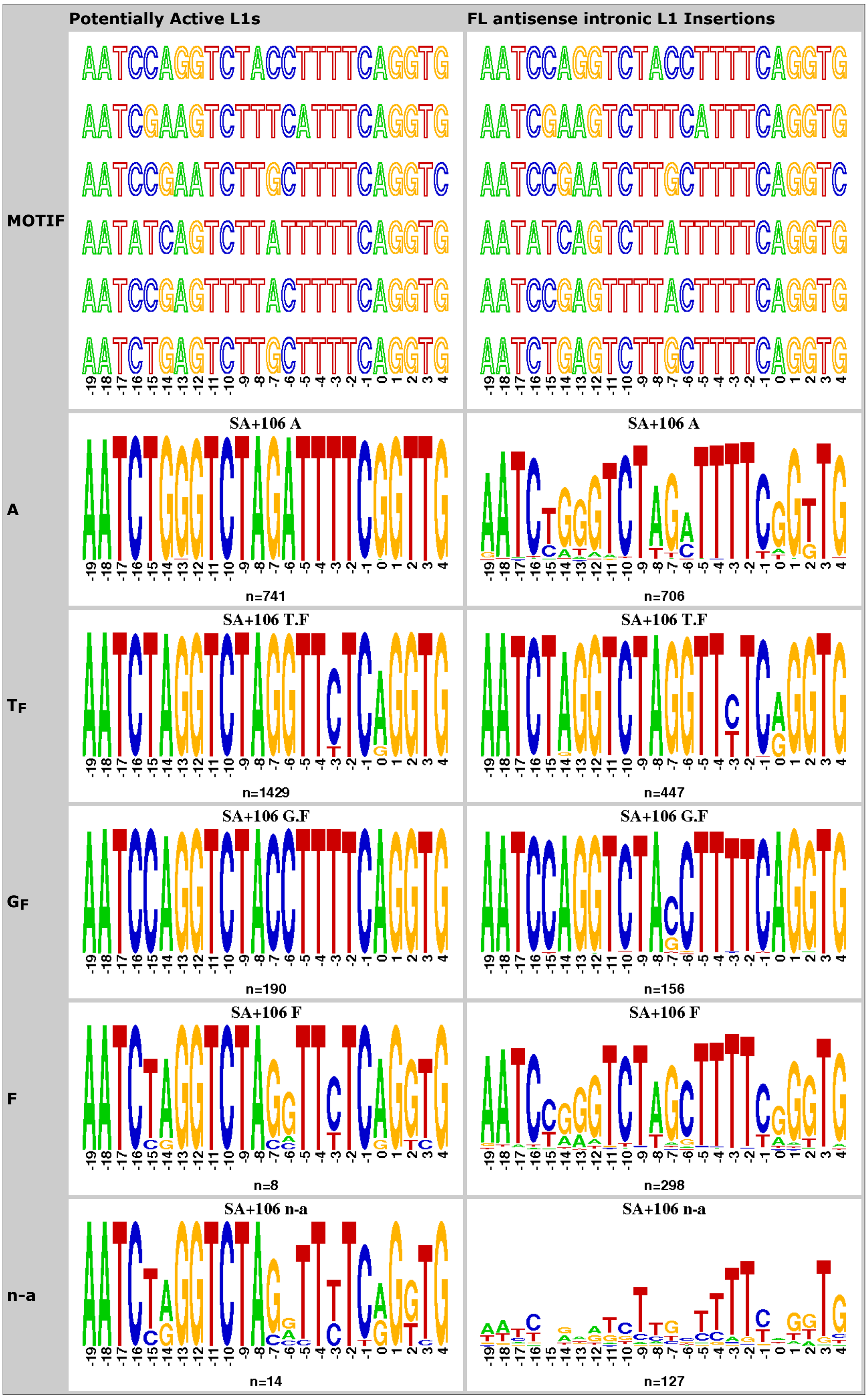

Potentially Active L1s
FL antisense intronic L1 Insertions
MOTIF
AATCCAGGTCTACCTTTTCAGGTG
AATCGAAGTCTTTCATTTCAGGTG
AATCCGAATCTTGCTTTTCAGGTC
AATATCAGTCTTATTTTTCAGGTG
AATCCGAGTTTTACTTTTCAGGTG
AATCTGAGTCTTGCTTTTCAGGTG
A
SA+106 A
n=741
n=706
TF
SA+106 T.F
n=1429
n=447
GF
SA+106 G.F
n=190
n=156
F
SA+106 F
n=8
n=298
n-a
SA+106 n-a
n=14
n=127

SA+120

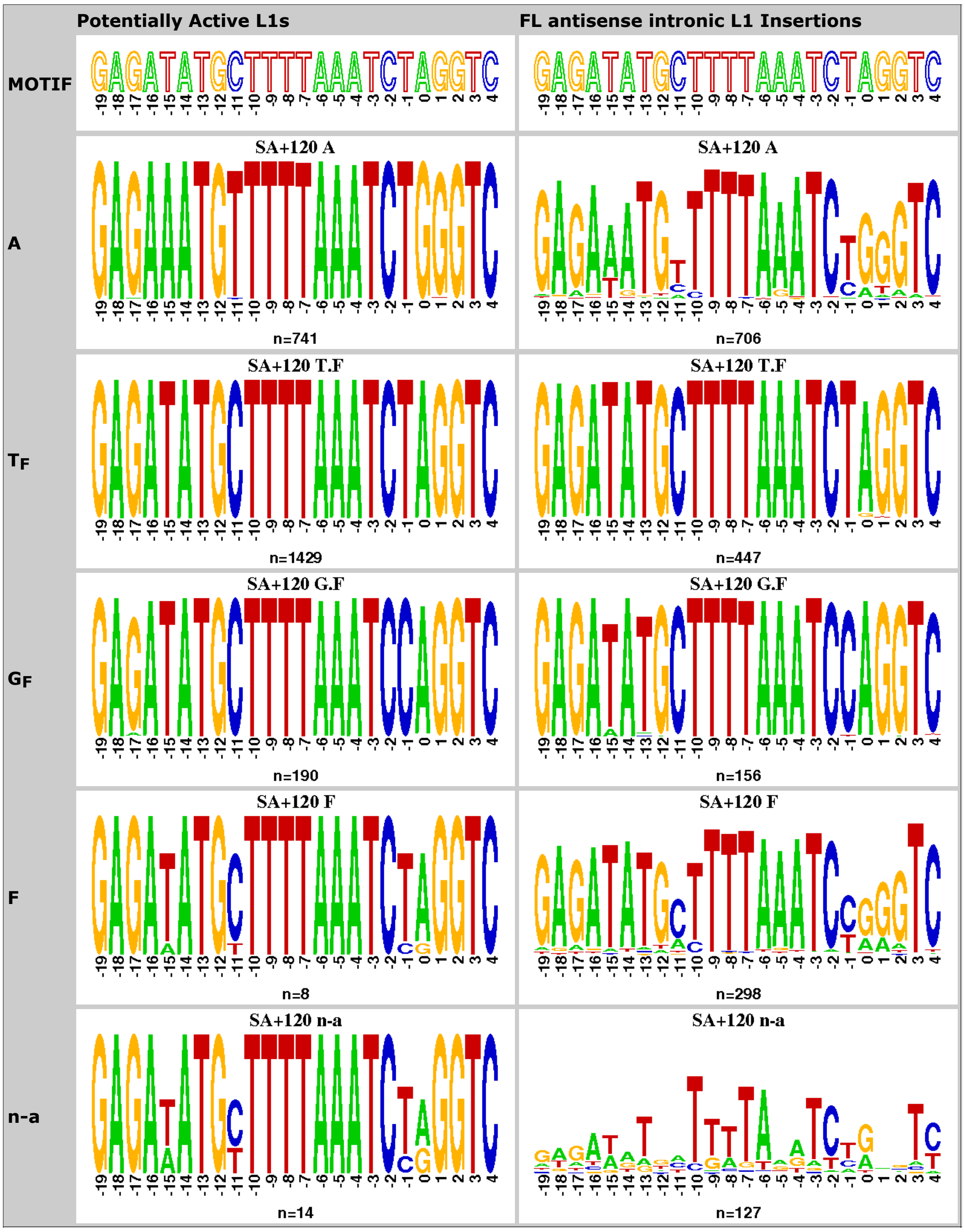

Potentially Active L1s
FL antisense intronic L1 Insertions
MOTIF
A
TF
GF
F
n-a
SA+120 A
n=741
SA+120 A
n=706
SA+120 T.F
n=1429
SA+120 T.F
n=447
SA+120 G.F
n=190
SA+120 G.F
n=156
SA+120 F
n=8
SA+120 F
n=298
SA+120 n-a
n=14
SA+120 n-a
n=127

SA+1930

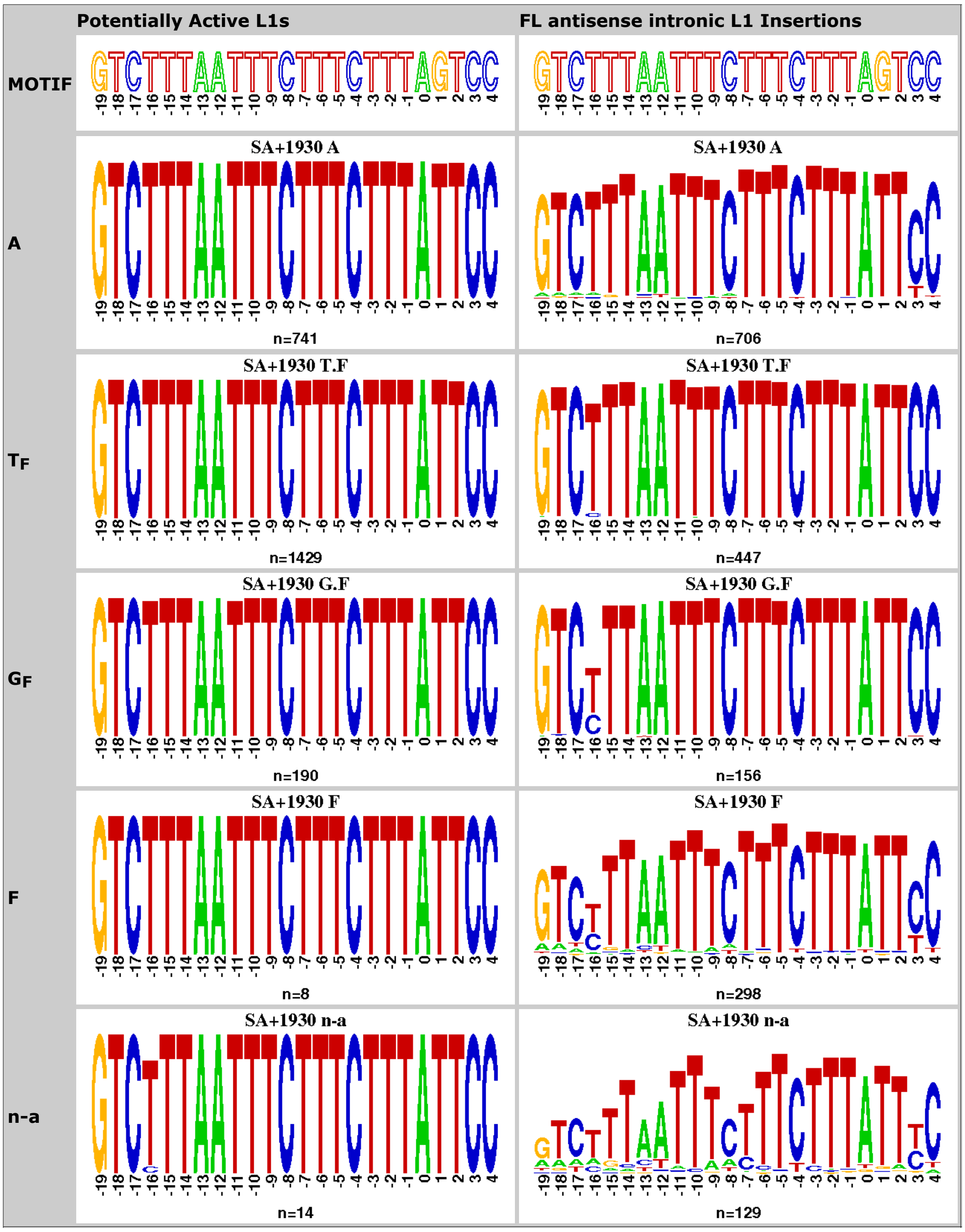

Potentially Active L1s
FL antisense intronic L1 Insertions
MOTIF
A
T F
G F
F
n-a
SA+1930 A
n=741
SA+1930 A
n=706
SA+1930 T.F
n=1429
SA+1930 T.F
n=447
SA+1930 G.F
n=190
SA+1930 G.F
n=156
SA+1930 F
n=8
SA+1930 F
n=298
SA+1930 n-a
n=14
SA+1930 n-a
n=129

SA+4117

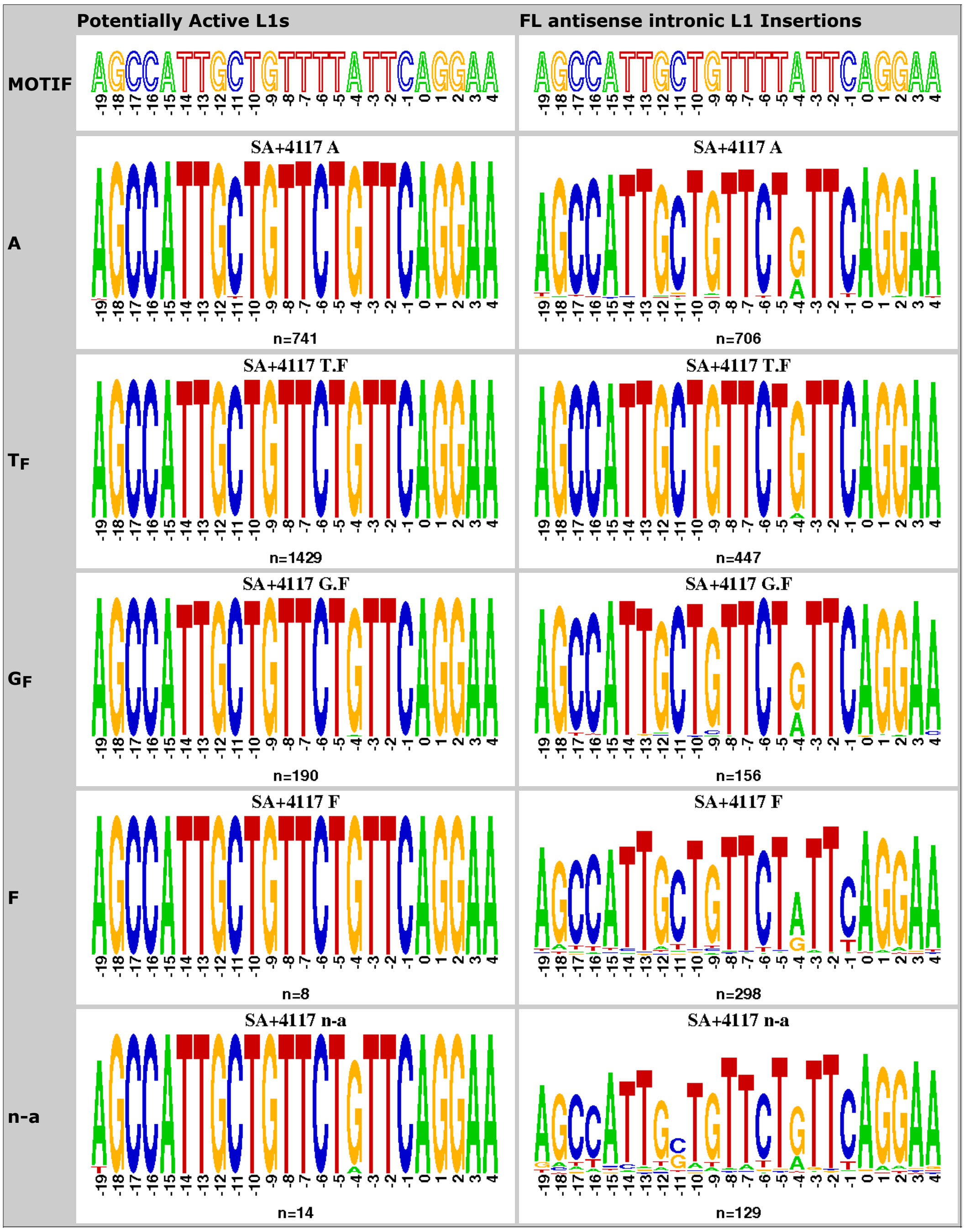
Potentially Active L1s
FL antisense intronic L1 Insertions
MOTIF
A
TF
GF
F
n-a
SA+4117 A
n=741
SA+4117 A
n=706
SA+4117 T.F
n=1429
SA+4117 T.F
n=447
SA+4117 G.F
n=190
SA+4117 G.F
n=156
SA+4117 F
n=8
SA+4117 F
n=298
SA+4117 n-a
n=14
SA+4117 n-a
n=129

SA+5260

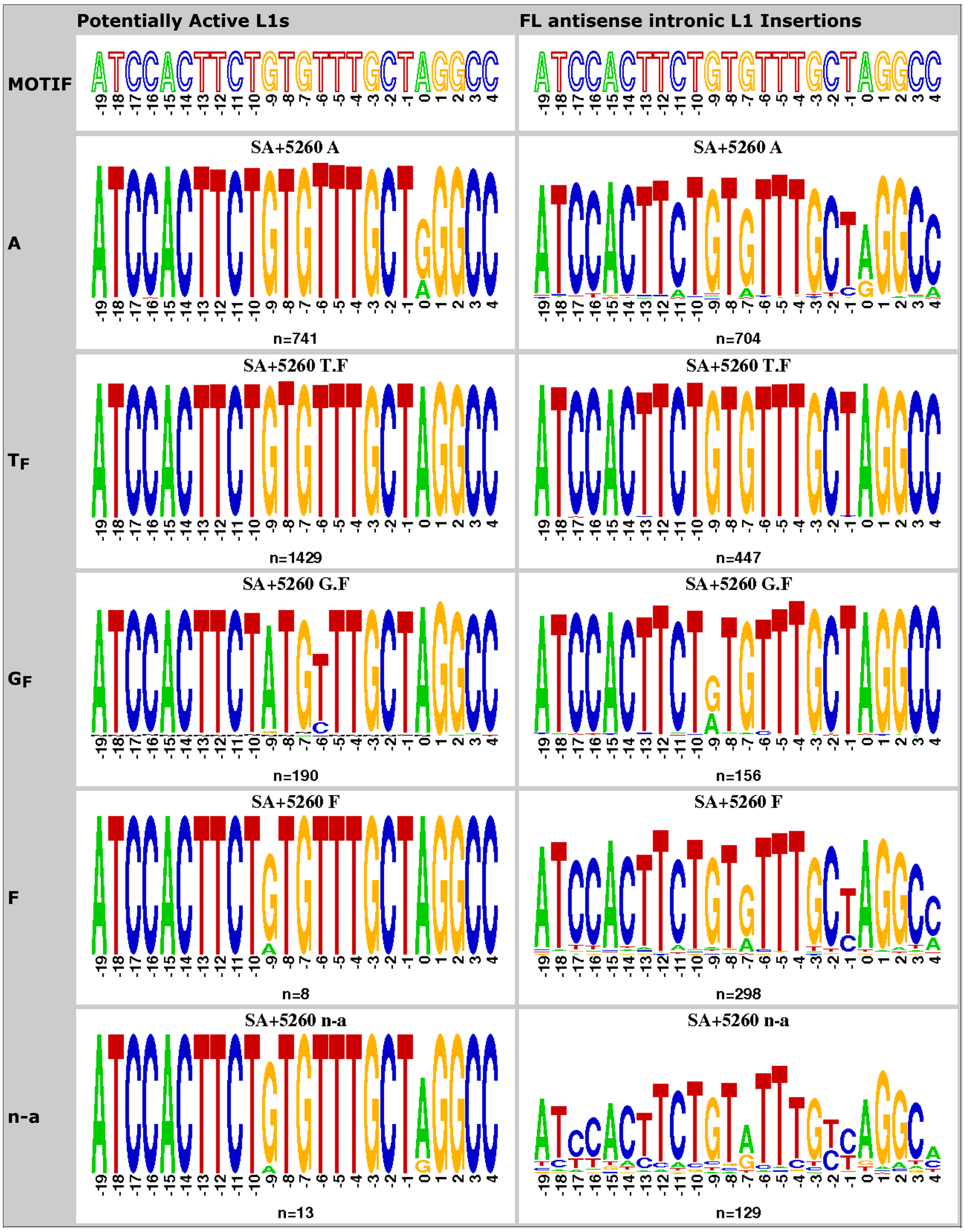

Potentially Active L1s
FL antisense intronic L1 Insertions
MOTIF
A
SA+5260 A
n=741
n=704
T_F
SA+5260 T.F
n=1429
n=447
G_F
SA+5260 G.F
n=190
n=156
F
SA+5260 F
n=8
n=298
n-a
SA+5260 n-a
n=13
n=129

SA+5614

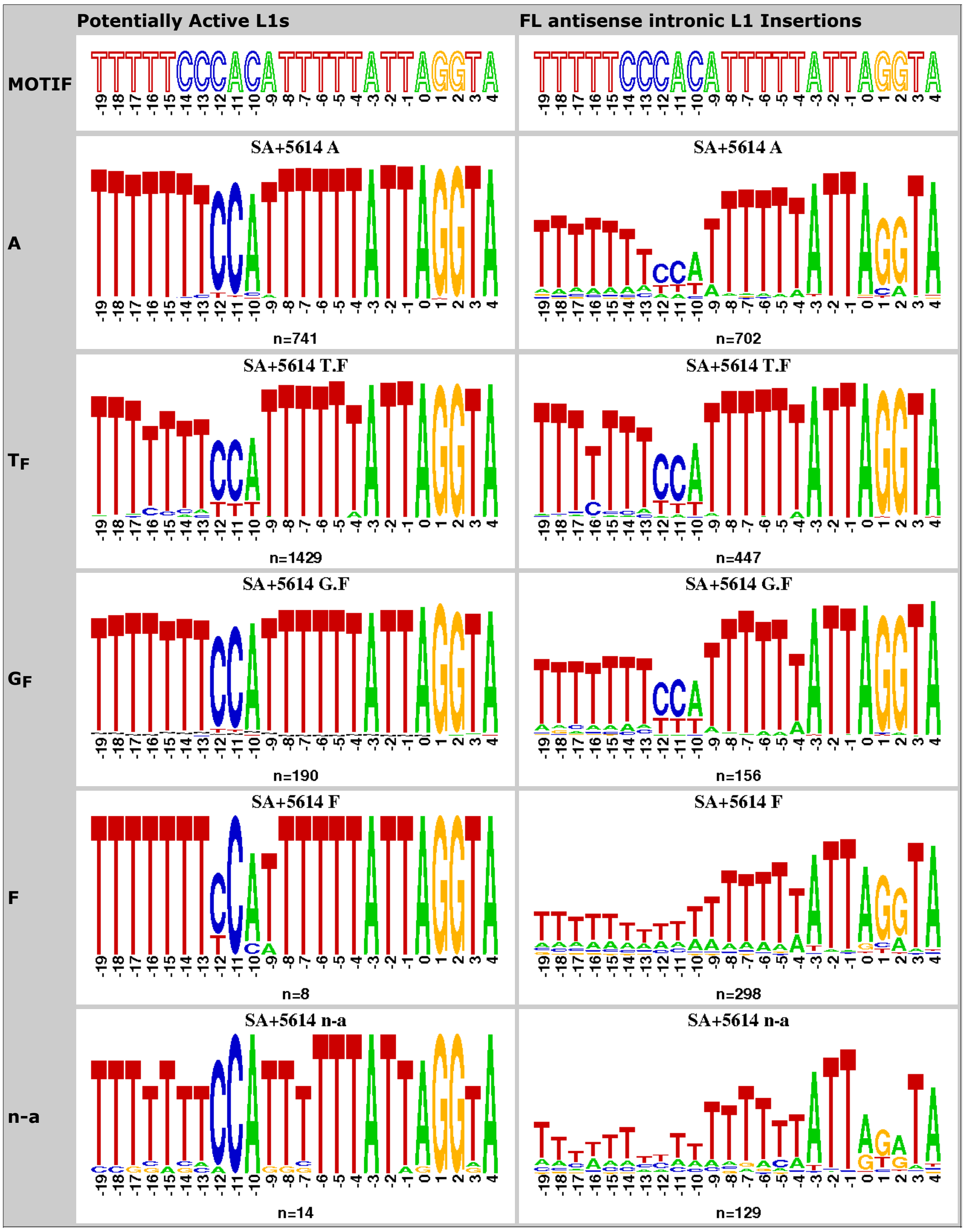

Potentially Active L1s
FL antisense intronic L1 Insertions
MOTIF
TTTTTCCCACATTTTTATTAGGTA
A
SA+5614 A
n=741
n=702
TF
SA+5614 T.F
n=1429
n=447
GF
SA+5614 G.F
n=190
n=156
F
SA+5614 F
n=8
n=298
n-a
SA+5614 n-a
n=14
n=129

SD+29

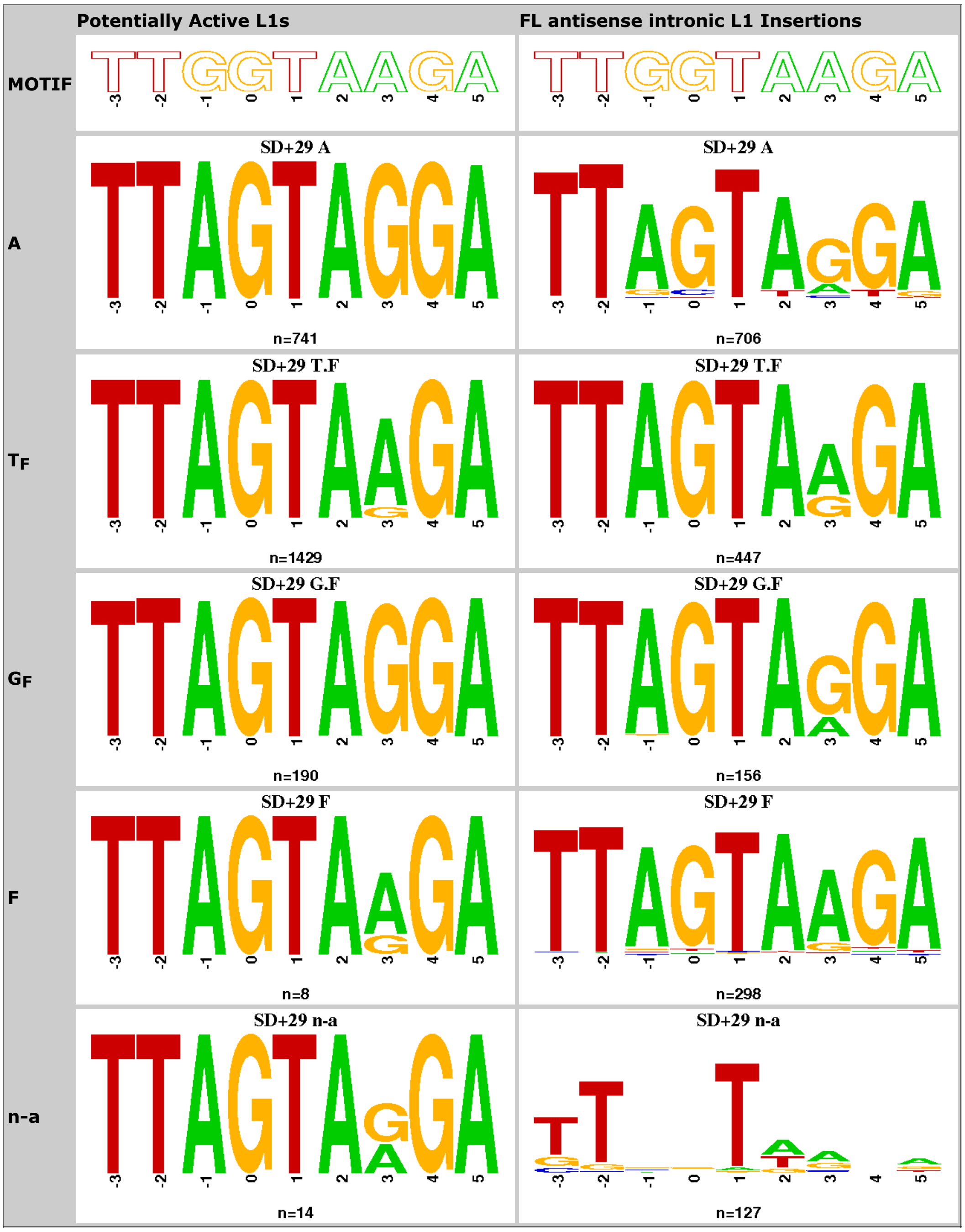
Potentially Active L1s
FL antisense intronic L1 Insertions
MOTIF
A
TF
GF
F
n-a
SD+29 A
n=741
SD+29 A
n=706
SD+29 T.F
n=1429
SD+29 T.F
n=447
SD+29 G.F
n=190
SD+29 G.F
n=156
SD+29 F
n=8
SD+29 F
n=298
SD+29 n-a
n=14
SD+29 n-a
n=127

SD+52

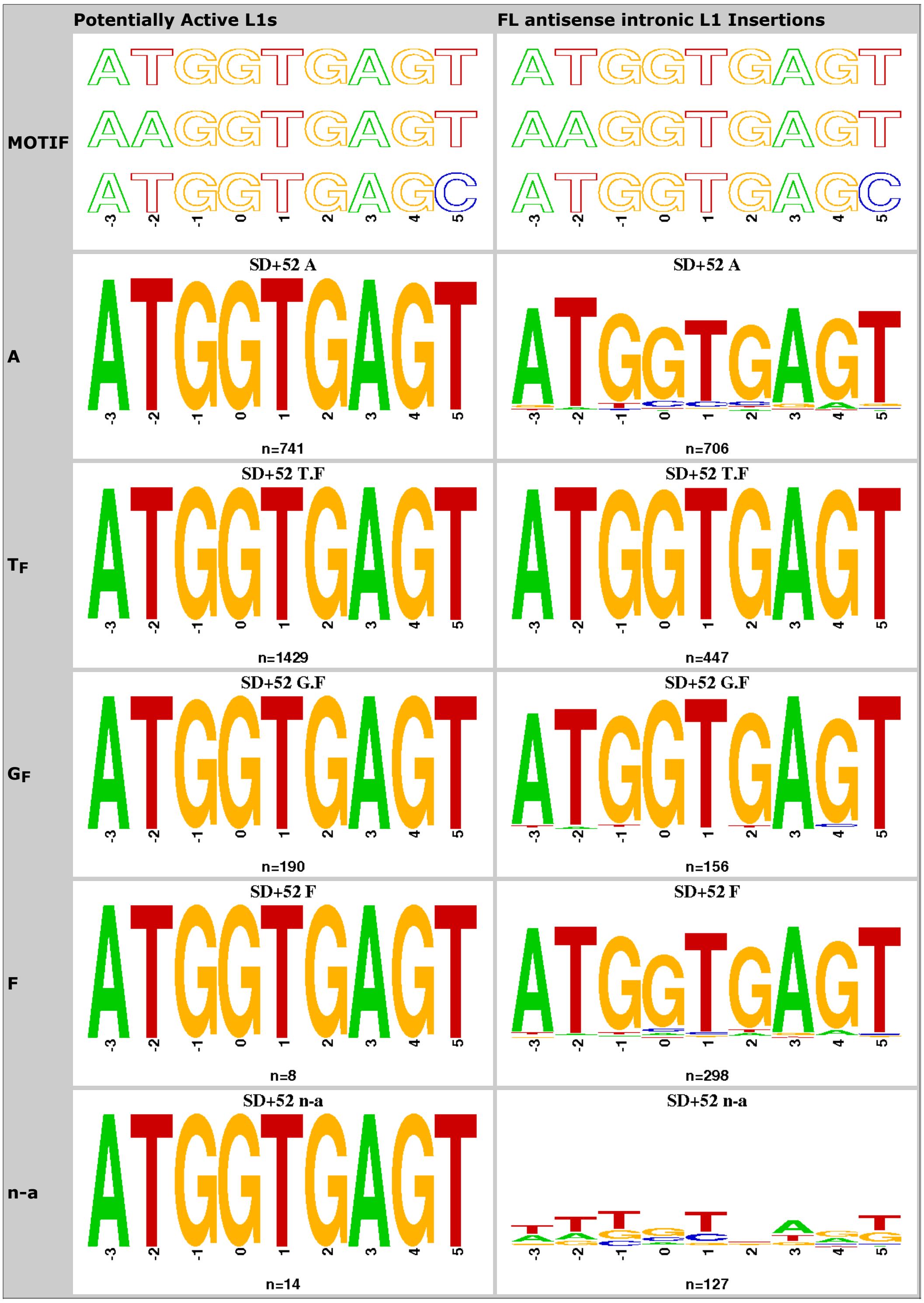

Potentially Active L1s
FL antisense intronic L1 Insertions
MOTIF
ATGGTGAGT
AAGGTGAGT
ATGGTGAGC
A
SD+52 A
n=741
SD+52 A
n=706
TF
SD+52 T.F
n=1429
SD+52 T.F
n=447
GF
SD+52 G.F
n=190
SD+52 G.F
n=156
F
SD+52 F
n=8
SD+52 F
n=298
n-a
SD+52 n-a
n=14
SD+52 n-a
n=127

SD+106

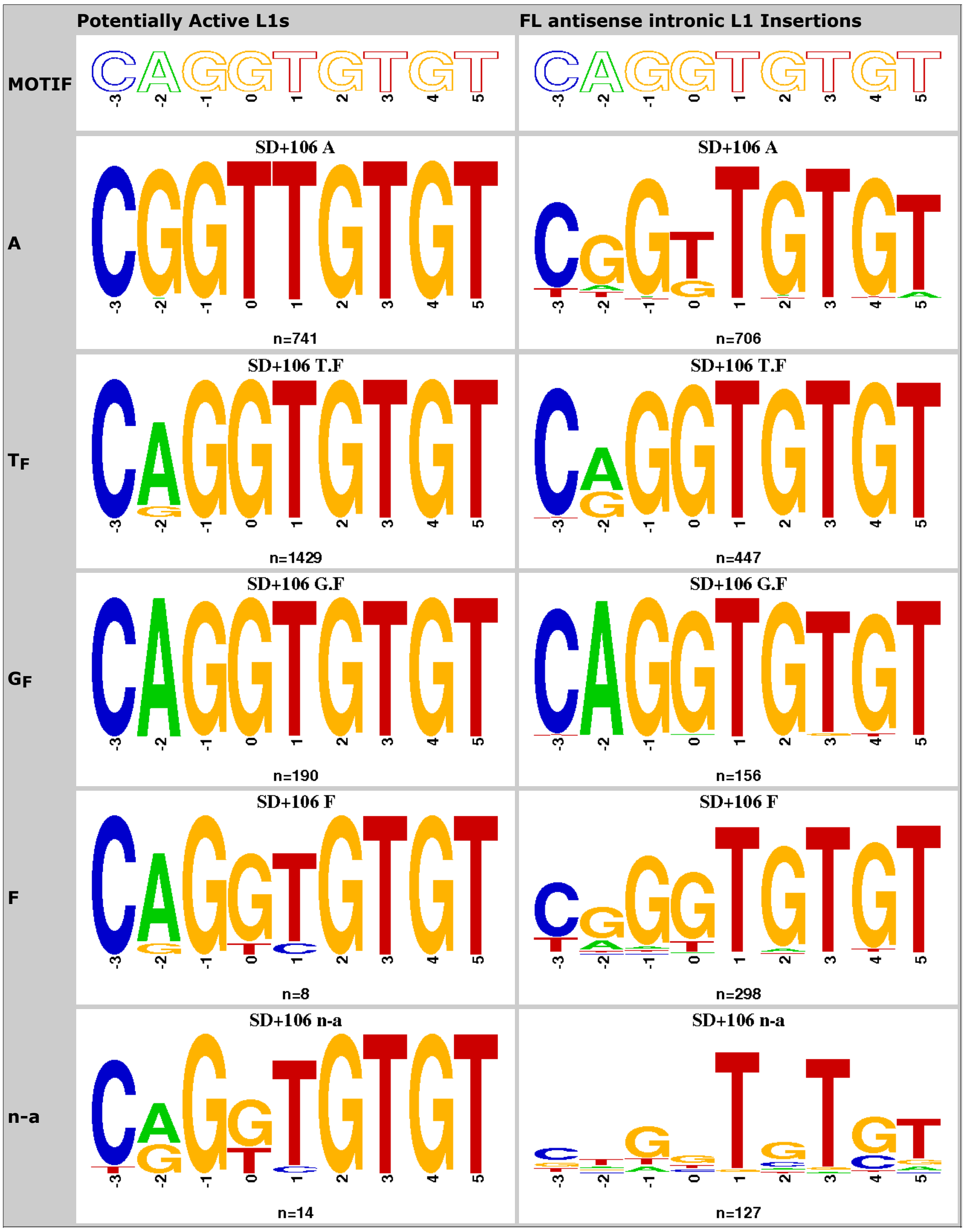

Potentially Active L1s
FL antisense intronic L1 Insertions
MOTIF
CAGGTGTGT
A
SD+106 A
n=741
n=706
TF
SD+106 T.F
n=1429
n=447
GF
SD+106 G.F
n=190
n=156
F
SD+106 F
n=8
n=298
n-a
SD+106 n-a
n=14
n=127
-3 -2 -1 0 1 2 3 4 5

SD+288

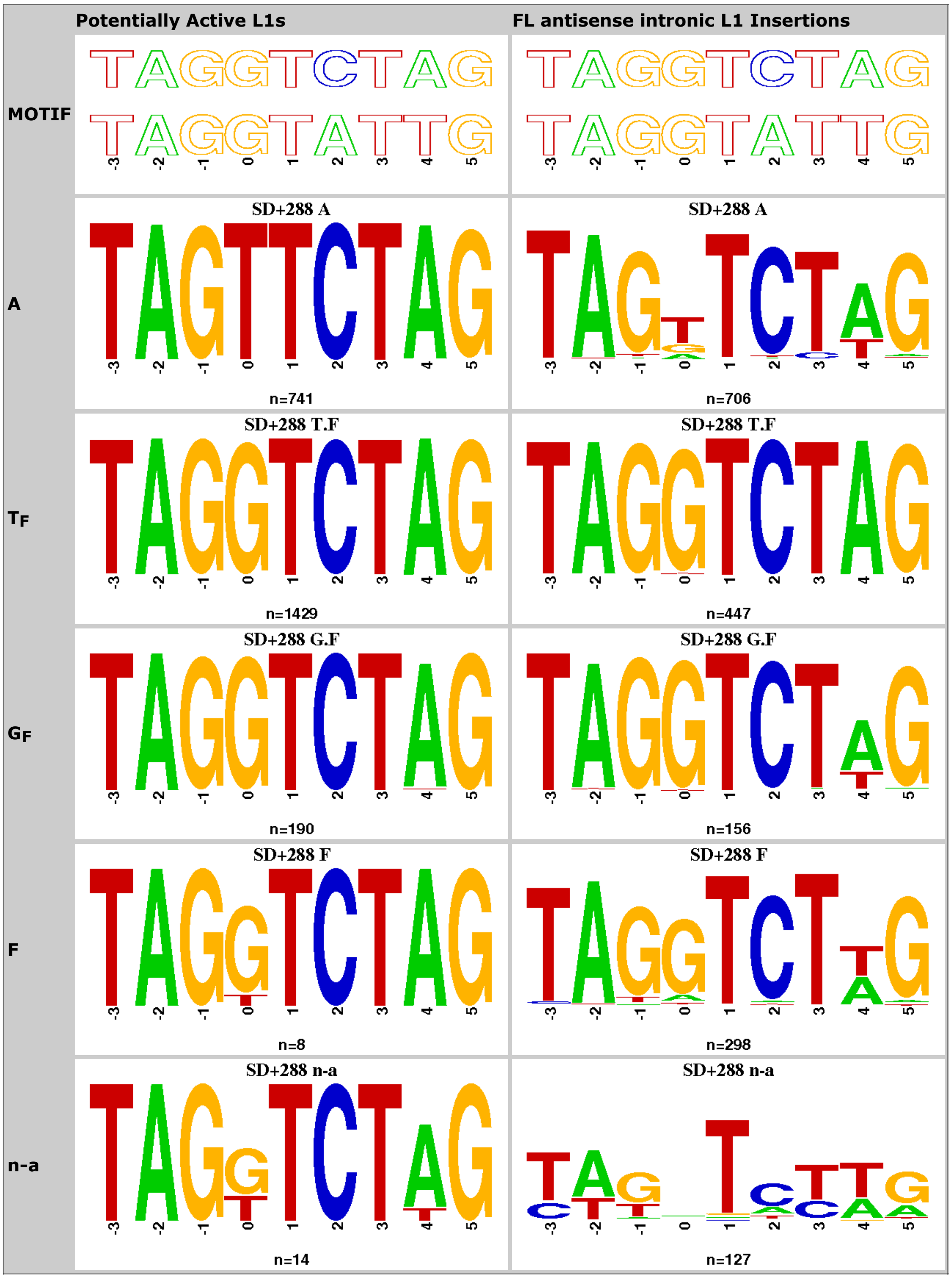

Potentially Active L1s
FL antisense intronic L1 Insertions
MOTIF
TAGGTCTAG
TAGGTATTG
A
TF
GF
F
n-a
SD+288 A
n=741
n=706
SD+288 T.F
n=1429
n=447
SD+288 G.F
n=190
n=156
SD+288 F
n=8
n=298
SD+288 n-a
n=14
n=127

SD+350

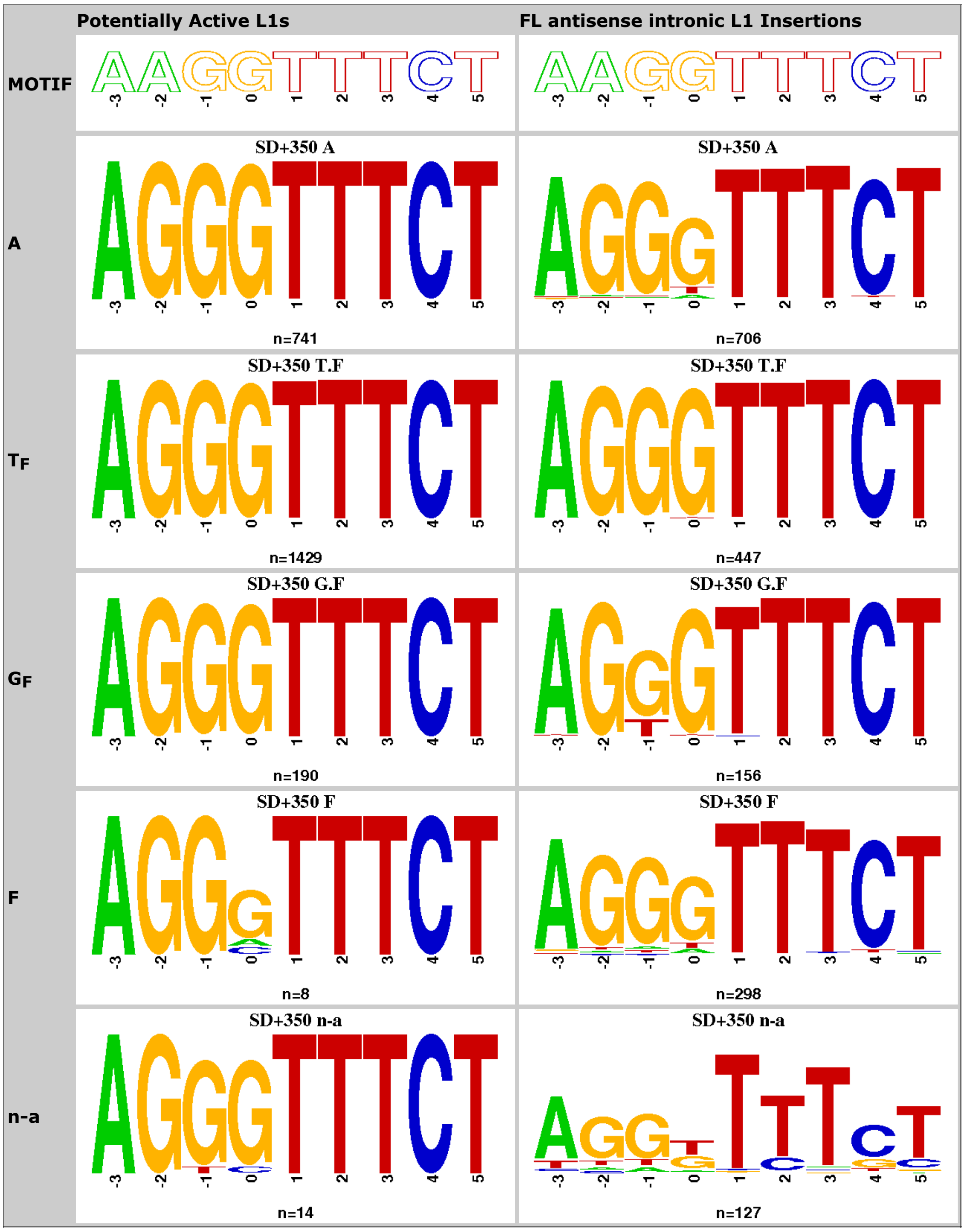
Potentially Active L1s
FL antisense intronic L1 Insertions
MOTIF
A
TF
GF
F
n-a
SD+350 A
n=741
SD+350 A
n=706
SD+350 T.F
n=1429
SD+350 T.F
n=447
SD+350 G.F
n=190
SD+350 G.F
n=156
SD+350 F
n=8
SD+350 F
n=298
SD+350 n-a
n=14
SD+350 n-a
n=127

SD+1881

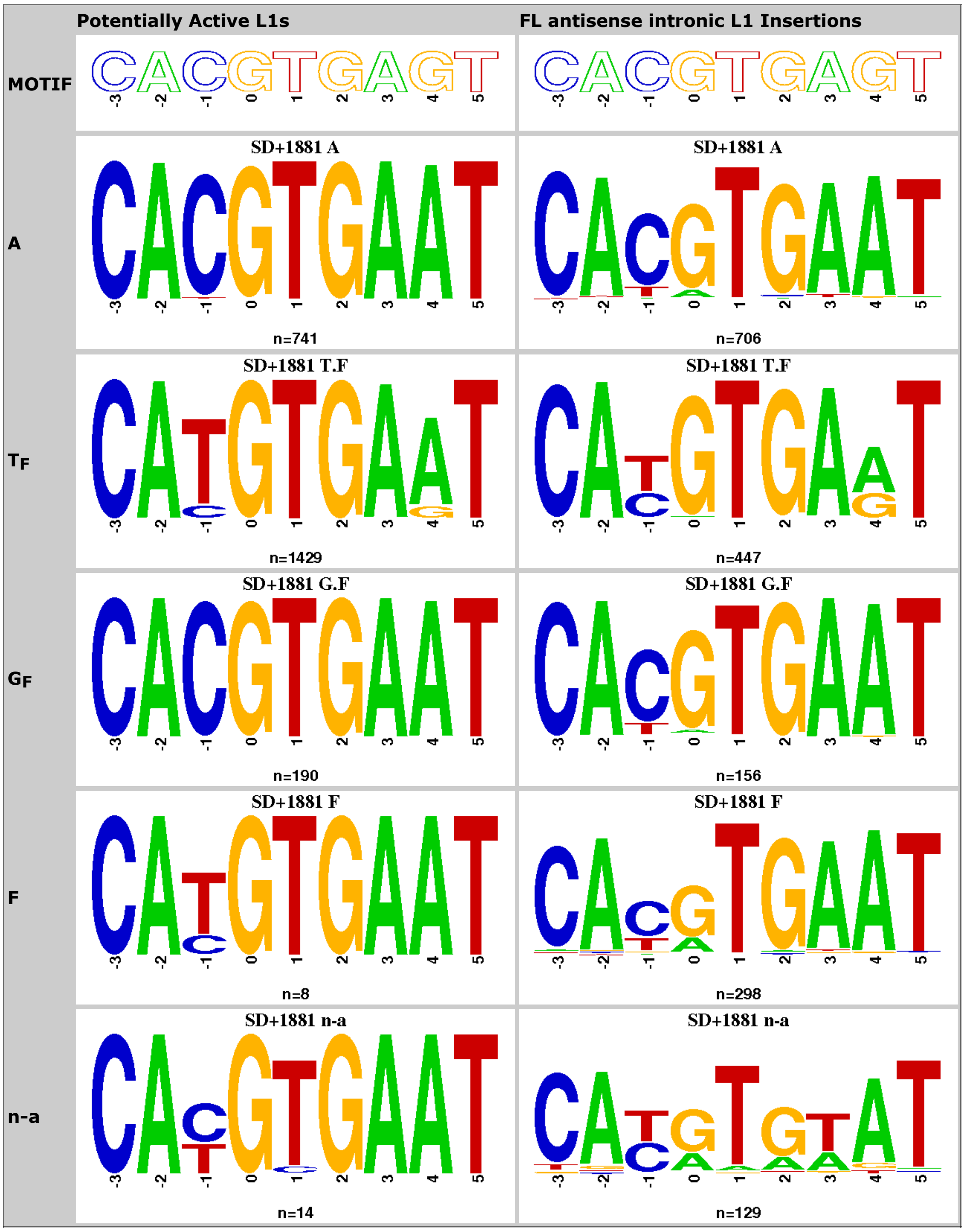
Potentially Active L1s
FL antisense intronic L1 Insertions
MOTIF
A
TF
GF
F
n-a
SD+1881 A
n=741
n=706
SD+1881 T.F
n=1429
n=447
SD+1881 G.F
n=190
n=156
SD+1881 F
n=8
n=298
SD+1881 n-a
n=14
n=129

SD+2036

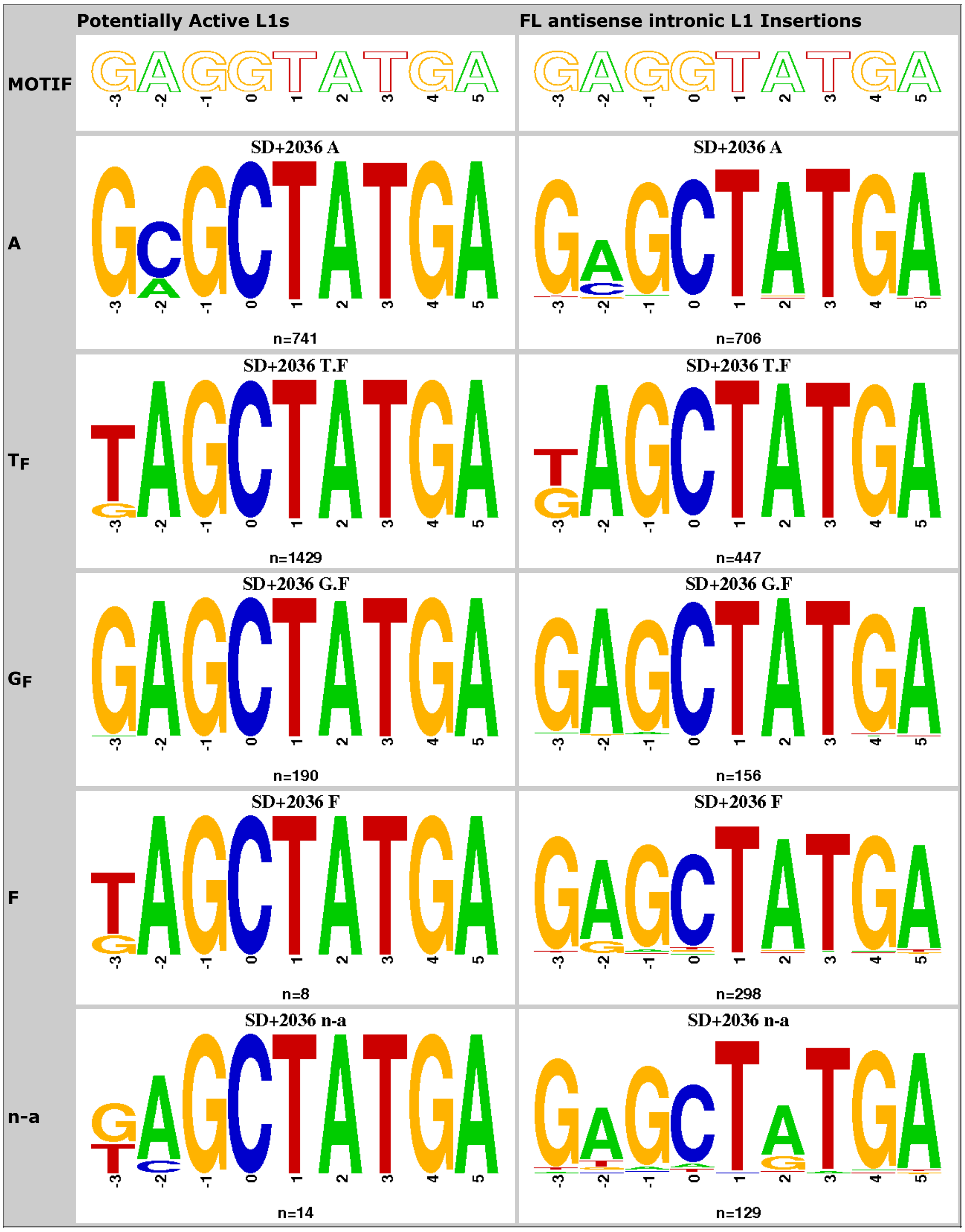

Potentially Active L1s
FL antisense intronic L1 Insertions
MOTIF
GAGGTATGA
GAGGTATGA
A
SD+2036 A
n=741
SD+2036 A
n=706
TF
SD+2036 T.F
n=1429
SD+2036 T.F
n=447
GF
SD+2036 G.F
n=190
SD+2036 G.F
n=156
F
SD+2036 F
n=8
SD+2036 F
n=298
n-a
SD+2036 n-a
n=14
SD+2036 n-a
n=129

SD+5094

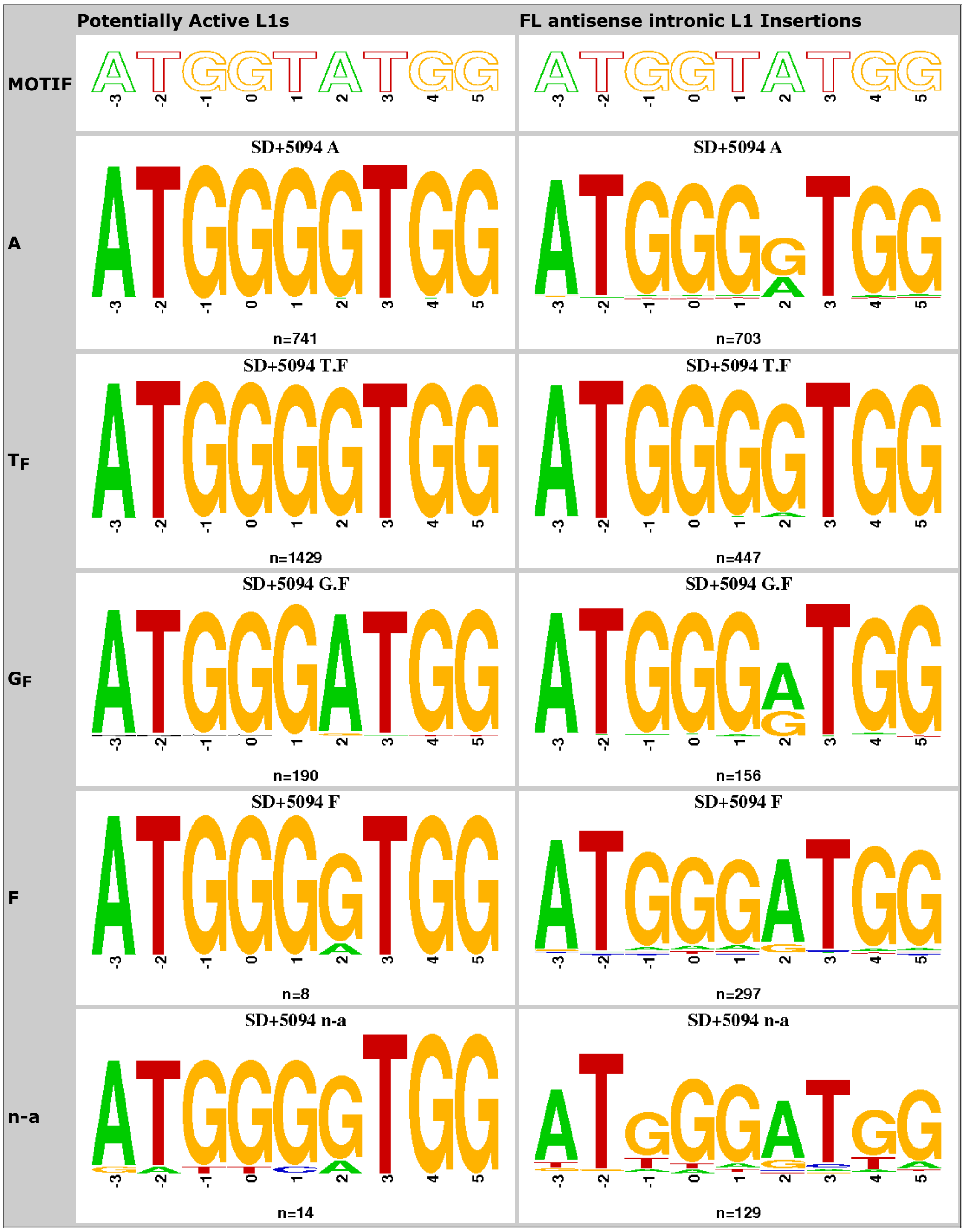

Potentially Active L1s
FL antisense intronic L1 Insertions
MOTIF
A
TF
GF
F
n-a
SD+5094 A
n=741
SD+5094 A
n=703
SD+5094 T.F
n=1429
SD+5094 T.F
n=447
SD+5094 G.F
n=190
SD+5094 G.F
n=156
SD+5094 F
n=8
SD+5094 F
n=297
SD+5094 n-a
n=14
SD+5094 n-a
n=129

A_SA+191

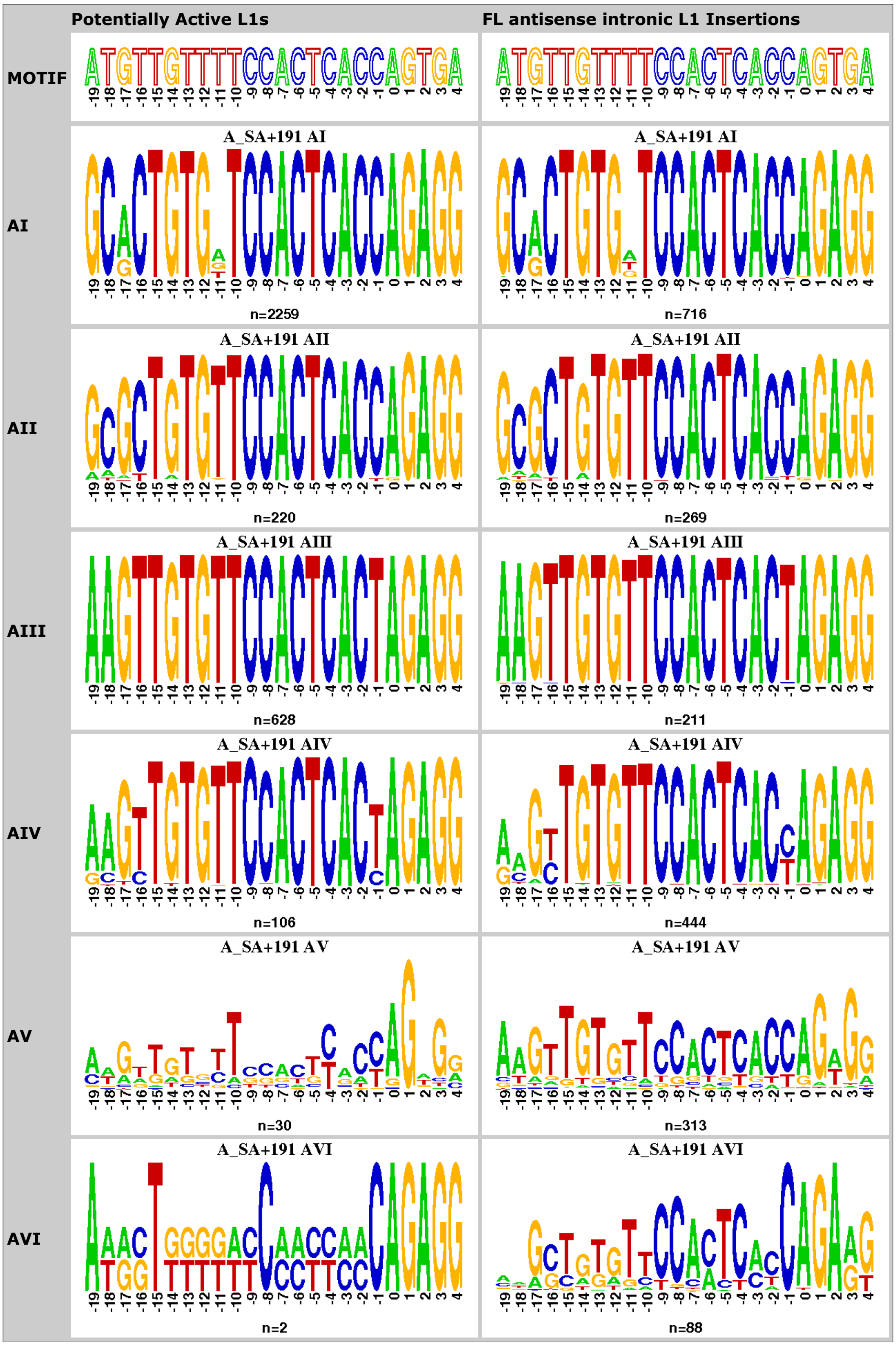
Potentially Active L1s
FL antisense intronic L1 Insertions
MOTIF
AI
AII
AIII
AIV
AV
AVI
A_SA+191 AI
n=2259
A_SA+191 AI
n=716
A_SA+191 AII
n=220
A_SA+191 AII
n=269
A_SA+191 AIII
n=628
A_SA+191 AIII
n=211
A_SA+191 AIV
n=106
A_SA+191 AIV
n=444
A_SA+191 AV
n=30
A_SA+191 AV
n=313
A_SA+191 AVI
n=2
A_SA+191 AVI
n=88

A_SD+72

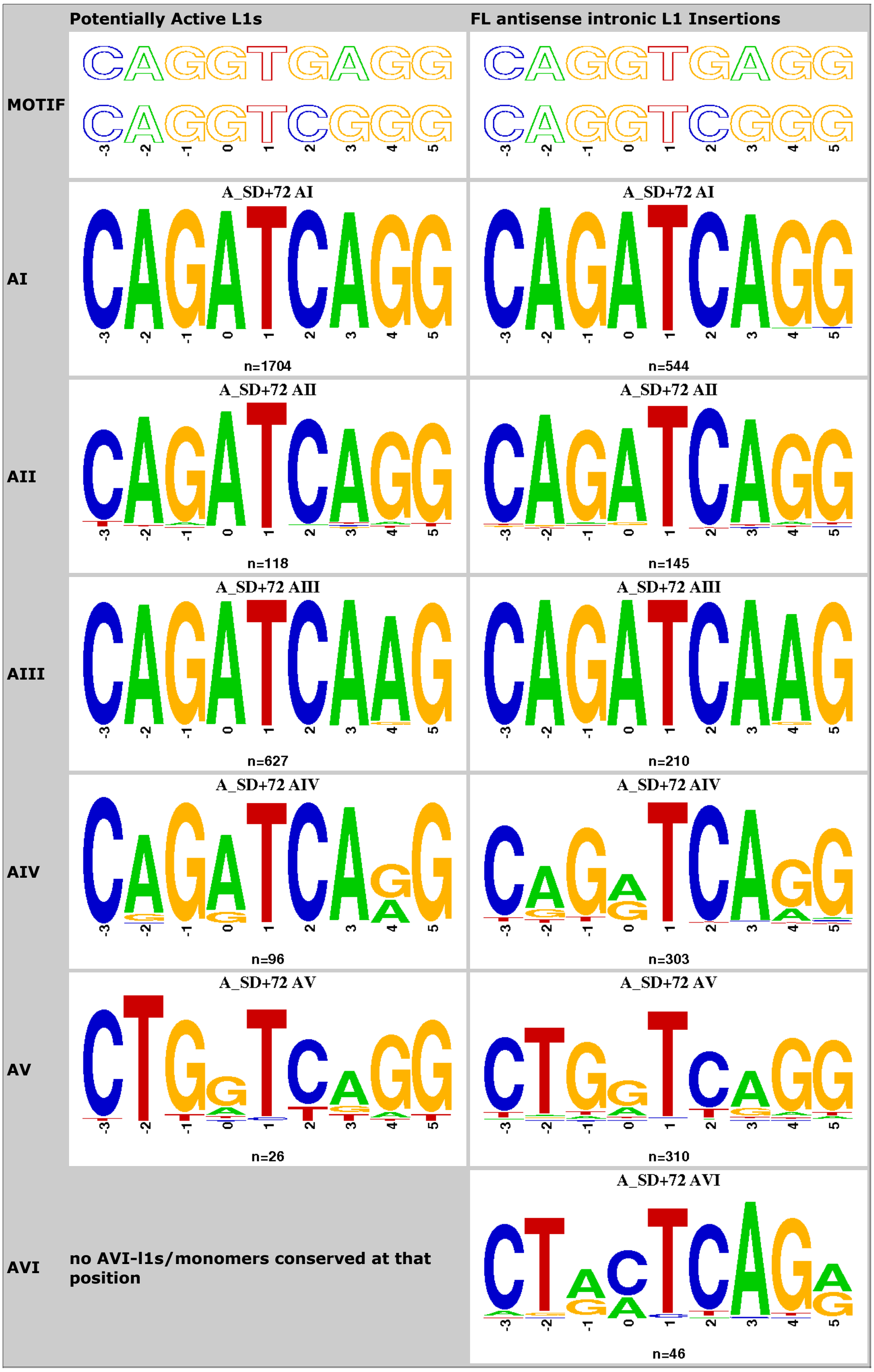
Potentially Active L1s
FL antisense intronic L1 Insertions
MOTIF
CAGGTGAGG
CAGGTCGGG
AI
A_SD+72 AI
n=1704
n=544
AII
A_SD+72 AII
n=118
n=145
AIII
A_SD+72 AIII
n=627
n=210
AIV
A_SD+72 AIV
n=96
n=303
AV
A_SD+72 AV
n=26
n=310
AVI
no AVI-l1s/monomers conserved at that position
A_SD+72 AVI
n=46

A_SD+122

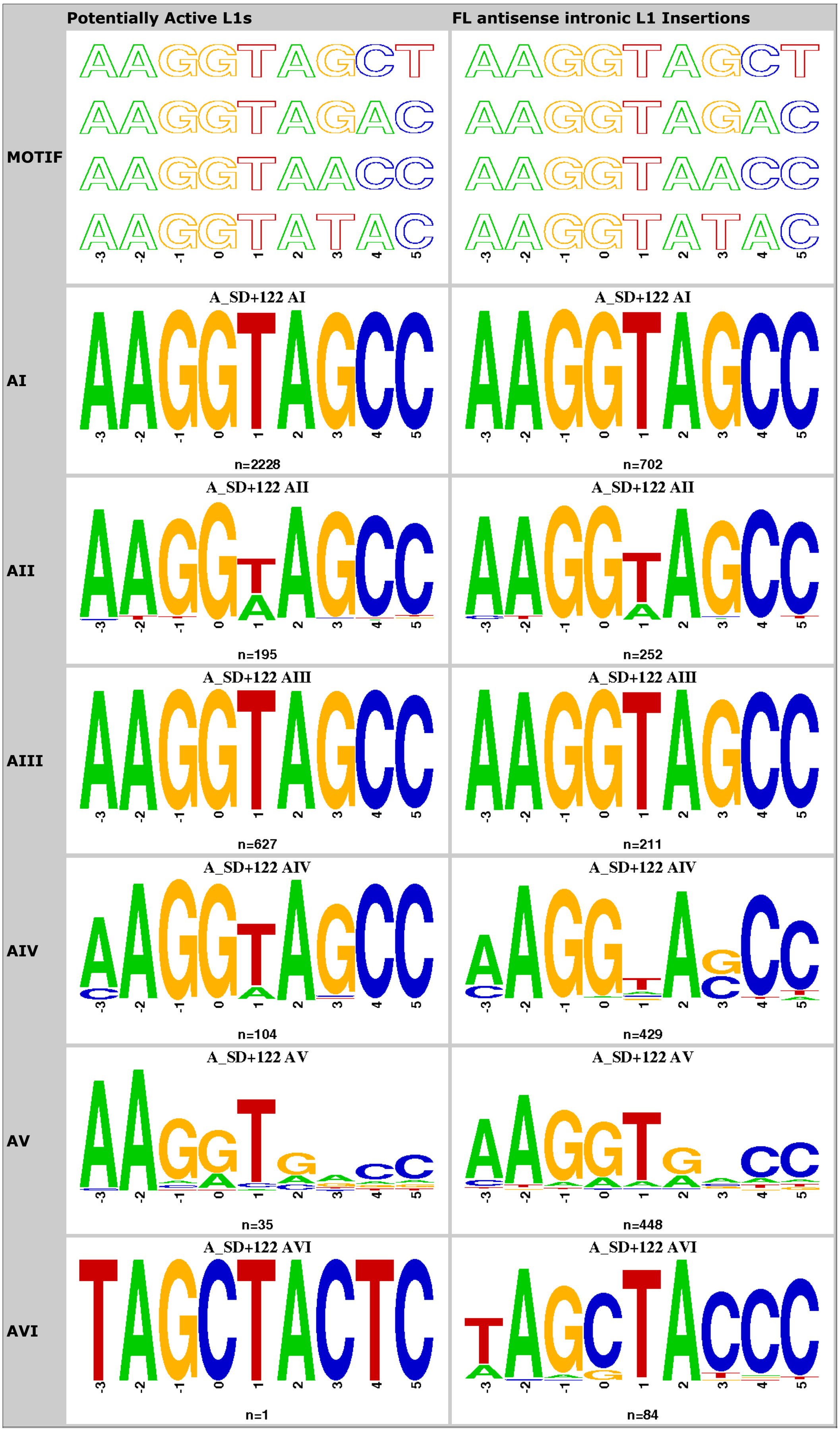
Potentially Active L1s
FL antisense intronic L1 Insertions
MOTIF
AAGGTAGCT
AAGGTAGAC
AAGGTAACC
AAGGTATAC
AI
A_SD+122 AI
n=2228
n=702
AII
A_SD+122 AII
n=195
n=252
AIII
A_SD+122 AIII
n=627
n=211
AIV
A_SD+122 AIV
n=104
n=429
AV
A_SD+122 AV
n=35
n=448
AVI
A_SD+122 AVI
n=1
n=84
-3 -2 -1 0 1 2 3 4 5

F_SA+100

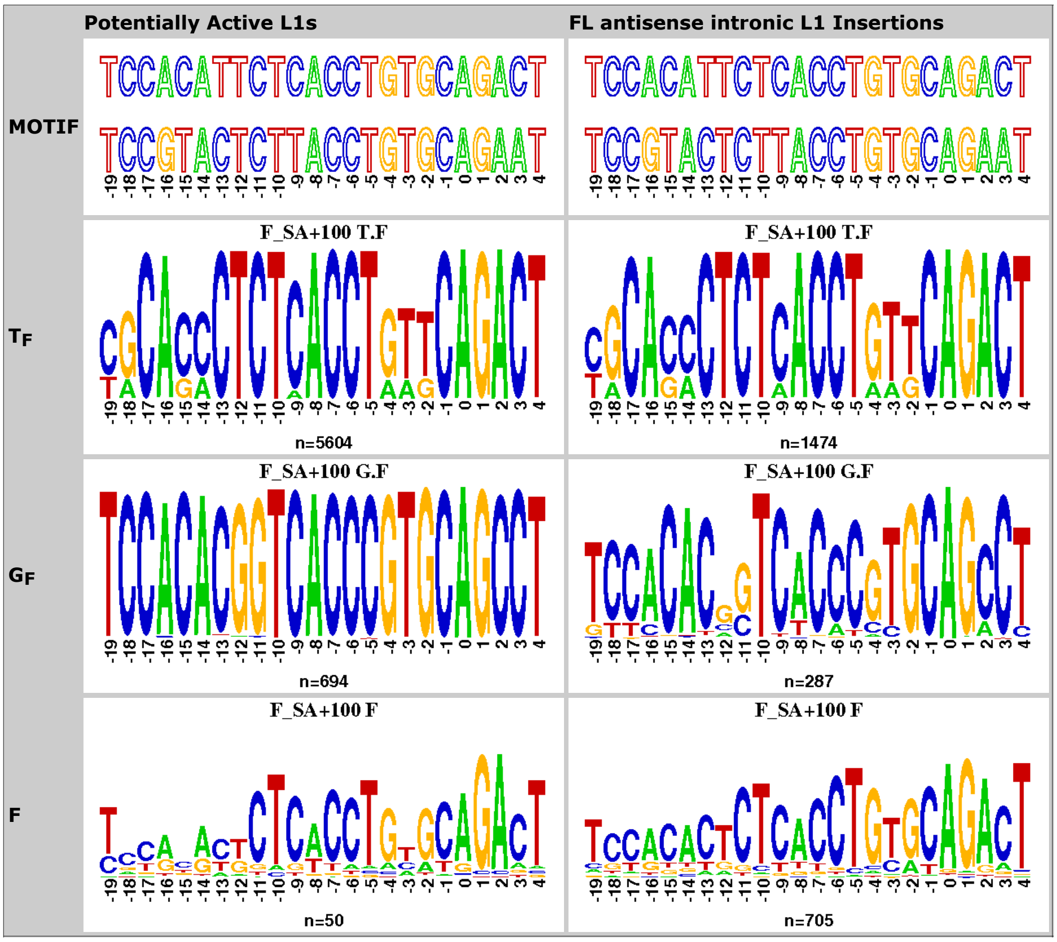
Potentially Active L1s
FL antisense intronic L1 Insertions
MOTIF
TCCACATTCTCACCTGTGCAGACT
TCCGTACTCTTACCTGTGCAGAAT
TF
F_SA+100 T.F
n=5604
n=1474
GF
F_SA+100 G.F
n=694
n=287
F
F_SA+100 F
n=50
n=705

F_SA+142

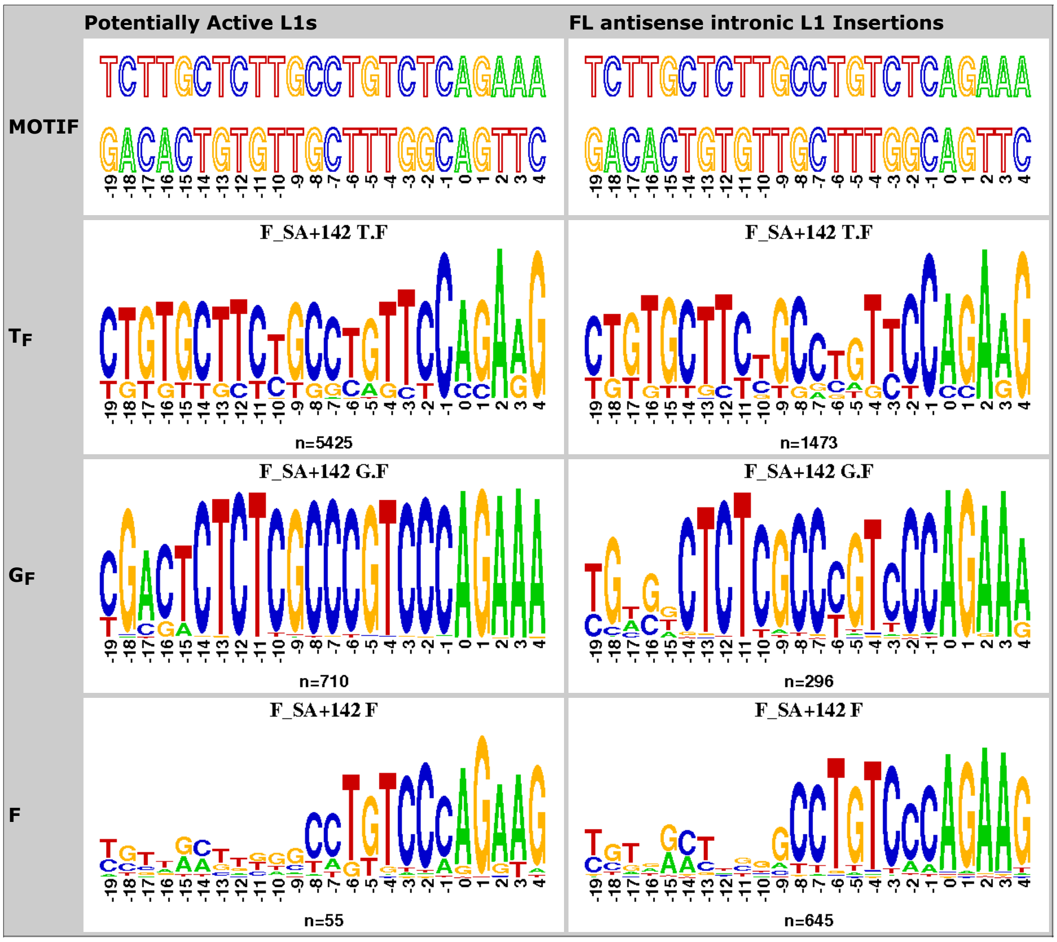

Potentially Active L1s
FL antisense intronic L1 Insertions
MOTIF
TCTTGCTCTTGCCTGTCTCAGAAA
GACACTGTGTTGCTTTGGCAGTTC
TF
F_SA+142 T.F
n=5425
n=1473
GF
F_SA+142 G.F
n=710
n=296
F
F_SA+142 F
n=55
n=645

F_SA+213

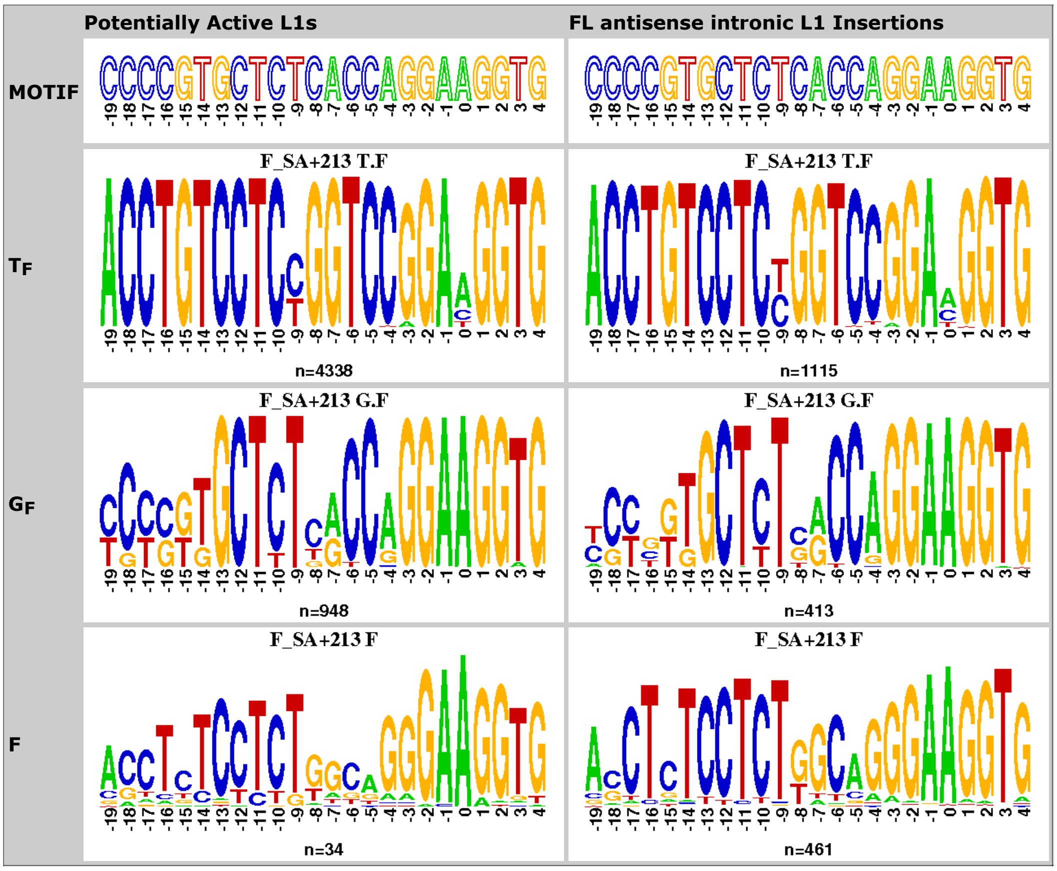

Potentially Active L1s
FL antisense intronic L1 Insertions
MOTIF
CCCCGTGCTCTCACCAGGAAGGTG
CCCCGTGCTCTCACCAGGAAGGTG
T_F
F_SA+213 T.F
n=4338
F_SA+213 T.F
n=1115
G_F
F_SA+213 G.F
n=948
F_SA+213 G.F
n=413
F
F_SA+213 F
n=34
F_SA+213 F
n=461

F_SA+218

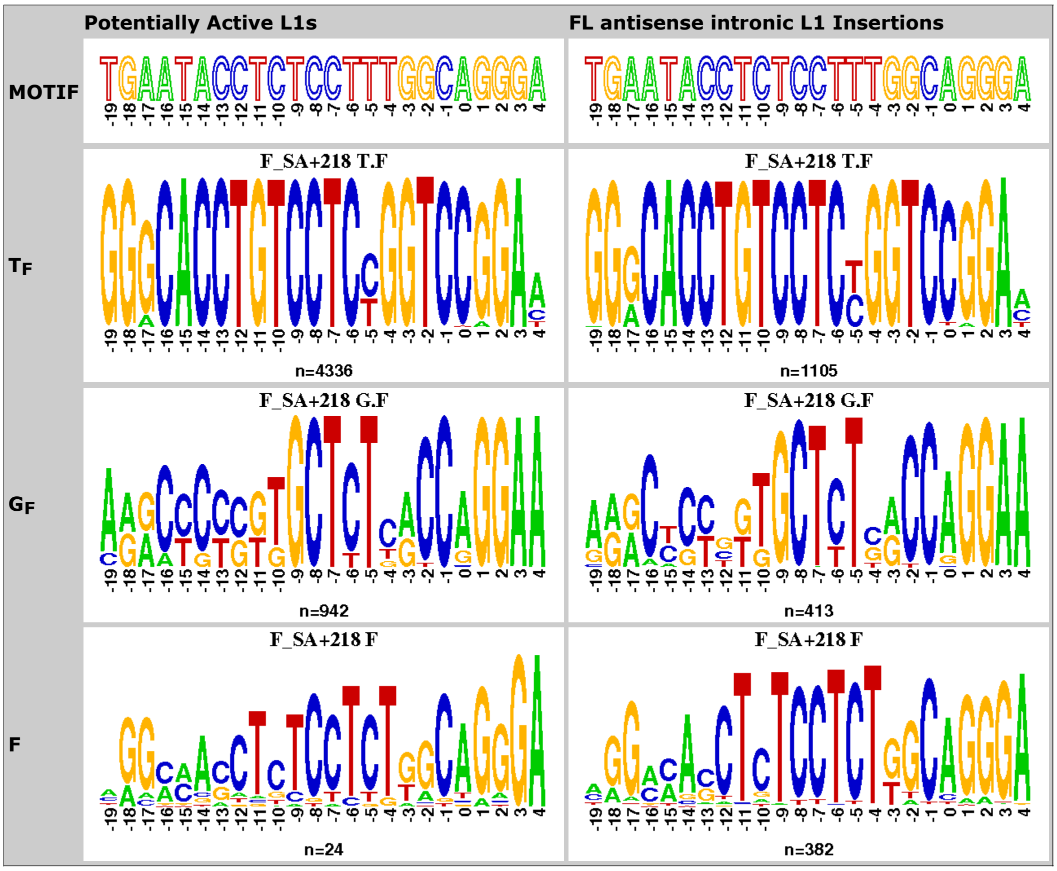

Potentially Active L1s
FL antisense intronic L1 Insertions
MOTIF
TGAATACCTCTCCTTTGGCAGGGA
TGAATACCTCTCCTTTGGCAGGGA
TF
F_SA+218 T.F
n=4336
F_SA+218 T.F
n=1105
GF
F_SA+218 G.F
n=942
F_SA+218 G.F
n=413
F
F_SA+218 F
n=24
F_SA+218 F
n=382

F_SD+24

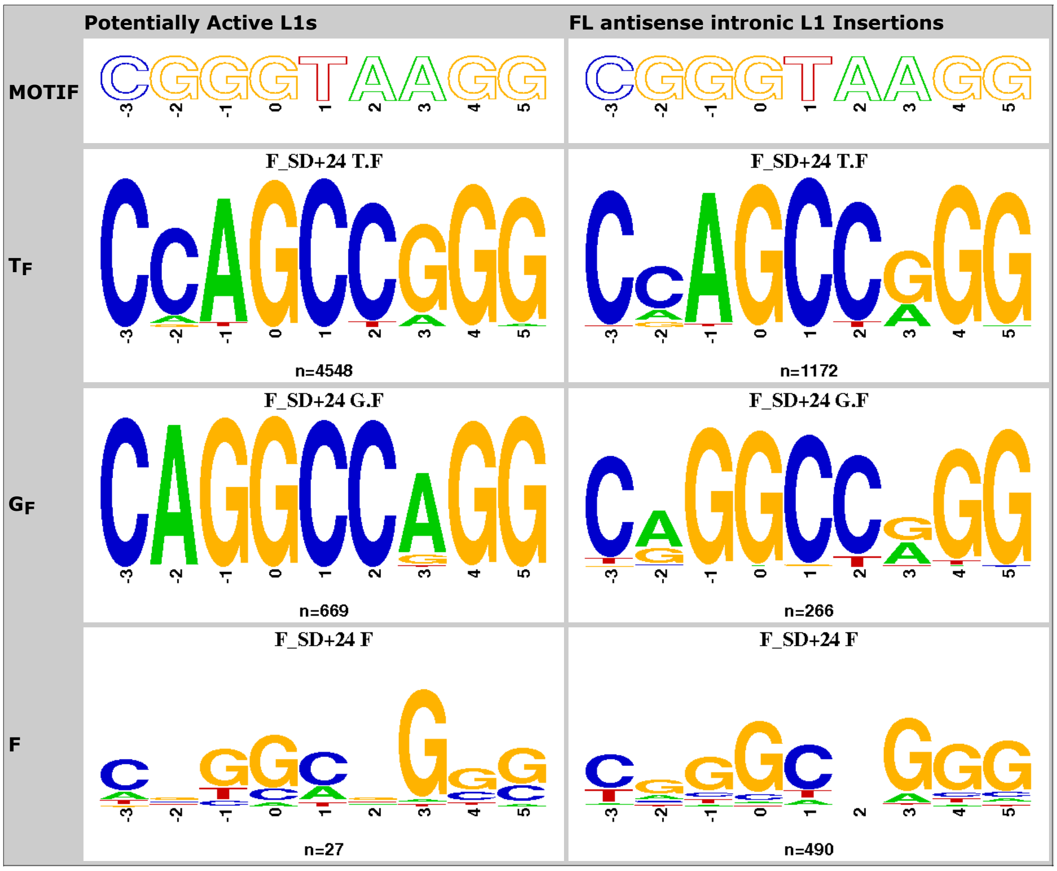

Potentially Active L1s
FL antisense intronic L1 Insertions
MOTIF
T_F
G_F
F
F_SD+24 T.F
n=4548
F_SD+24 T.F
n=1172
F_SD+24 G.F
n=669
F_SD+24 G.F
n=266
F_SD+24 F
n=27
F_SD+24 F
n=490

F_SD+213

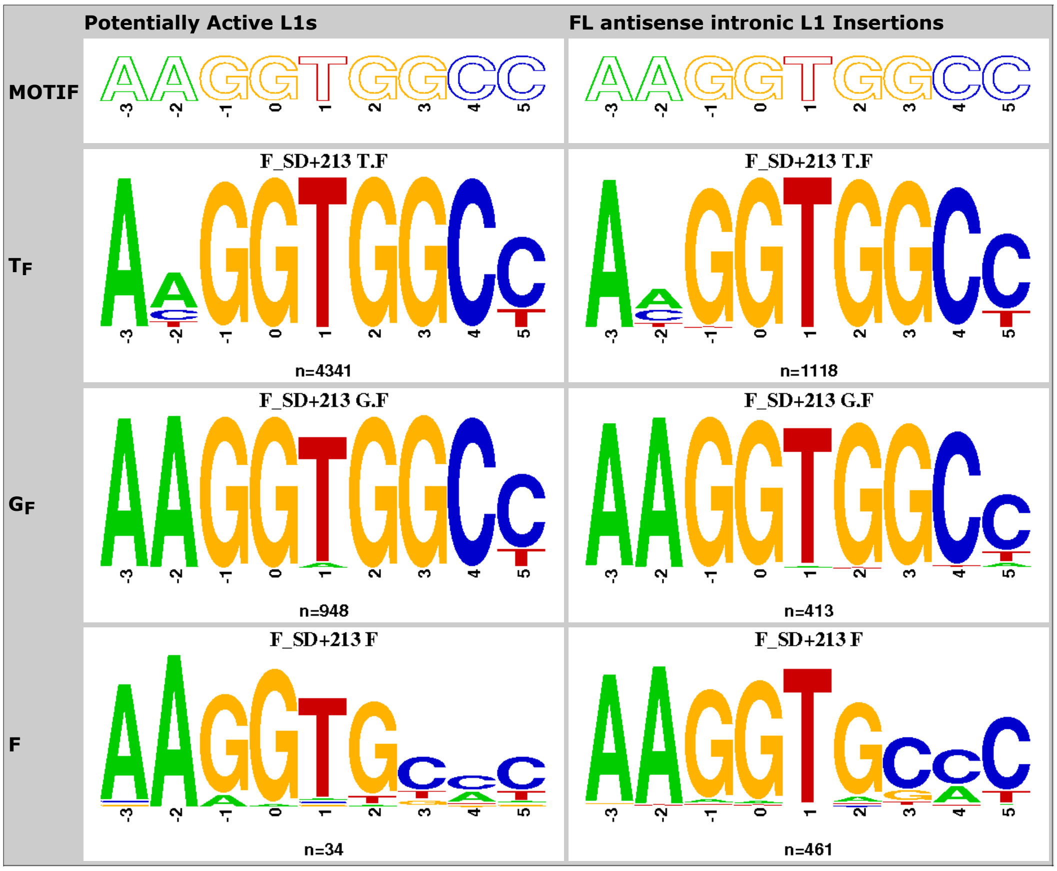
Potentially Active L1s
FL antisense intronic L1 Insertions
MOTIF
TF
GF
F
F_SD+213 T.F
n=4341
F_SD+213 T.F
n=1118
F_SD+213 G.F
n=948
F_SD+213 G.F
n=413
F_SD+213 F
n=34
F_SD+213 F
n=461

F_SD+218

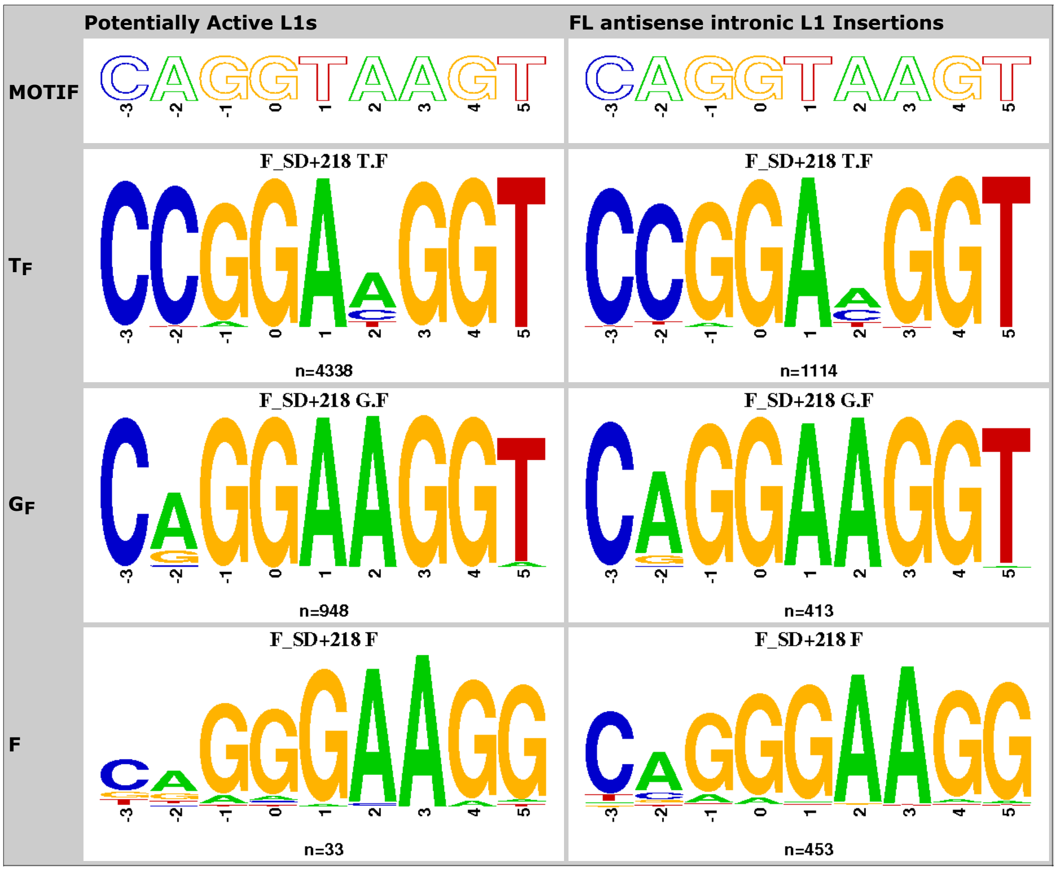
Potentially Active L1s
FL antisense intronic L1 Insertions
MOTIF
CAGGTAAGT
CAGGTAAGT
-3 -2 -1 0 1 2 3 4 5
T_F
F_SD+218 T.F
n=4338
F_SD+218 T.F
n=1114
G_F
F_SD+218 G.F
n=948
F_SD+218 G.F
n=413
F
F_SD+218 F
n=33
F_SD+218 F
n=453

S_SA+1237

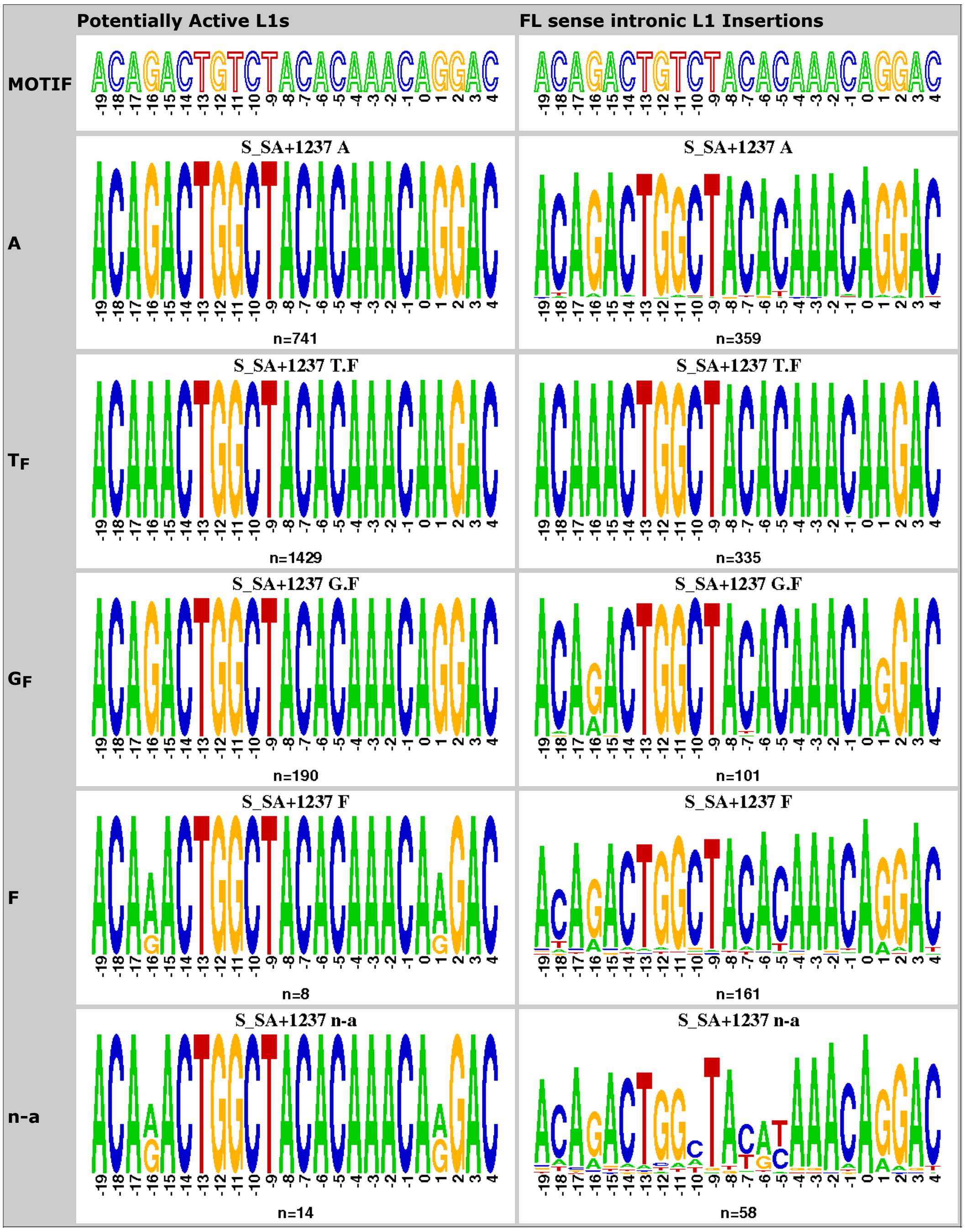

Potentially Active L1s
FL sense intronic L1 Insertions
MOTIF
ACAGACTGTCTACACAAACAGGAC
A
S_SA+1237 A
n=741
n=359
TF
S_SA+1237 T.F
n=1429
n=335
GF
S_SA+1237 G.F
n=190
n=101
F
S_SA+1237 F
n=8
n=161
n-a
S_SA+1237 n-a
n=14
n=58

BG_SA+4578

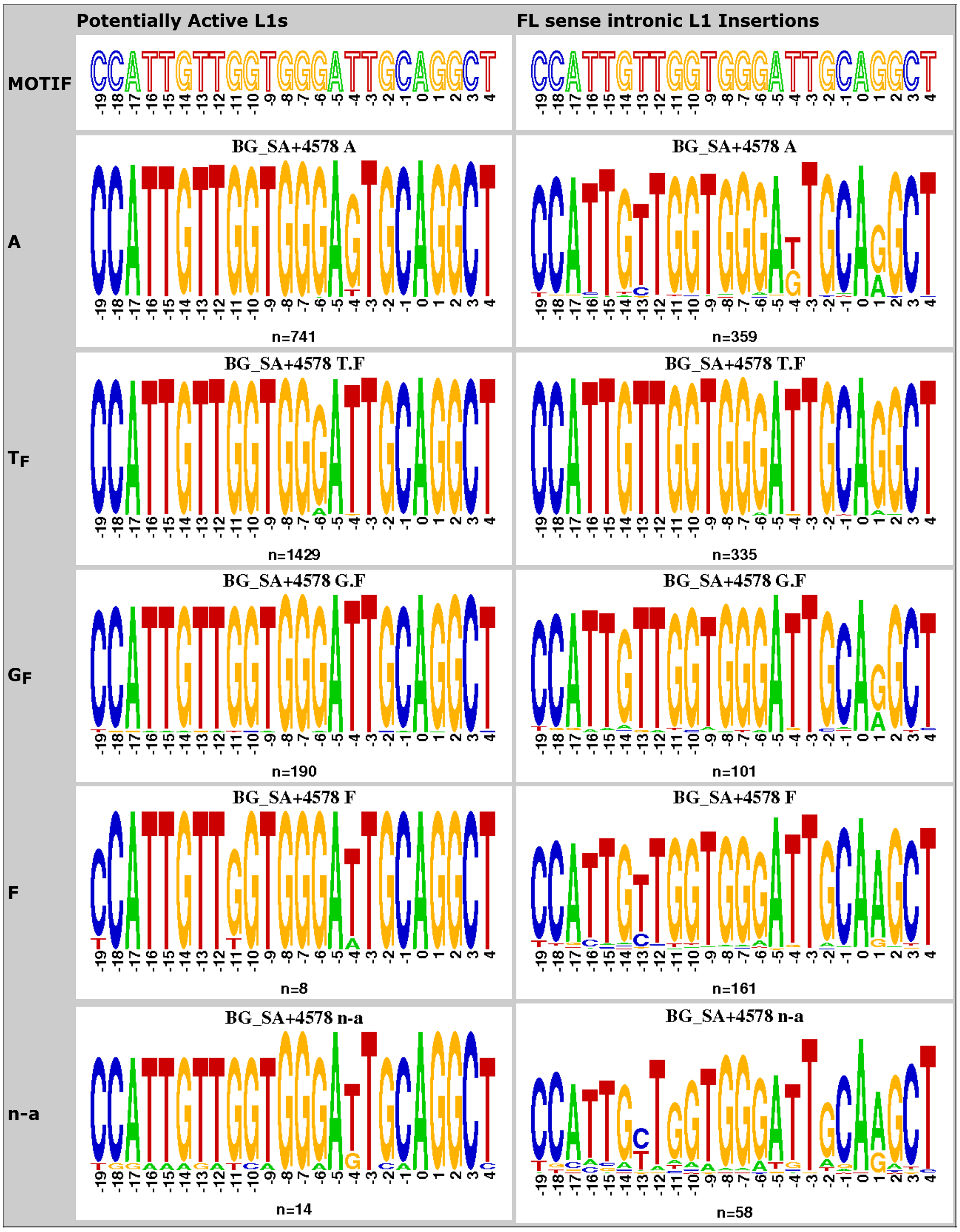

Potentially Active L1s
FL sense intronic L1 Insertions
MOTIF
A
TF
GF
F
n-a
BG_SA+4578 A
n=741
BG_SA+4578 A
n=359
BG_SA+4578 T.F
n=1429
BG_SA+4578 T.F
n=335
BG_SA+4578 G.F
n=190
BG_SA+4578 G.F
n=101
BG_SA+4578 F
n=8
BG_SA+4578 F
n=161
BG_SA+4578 n-a
n=14
BG_SA+4578 n-a
n=58

BG_SD+4694

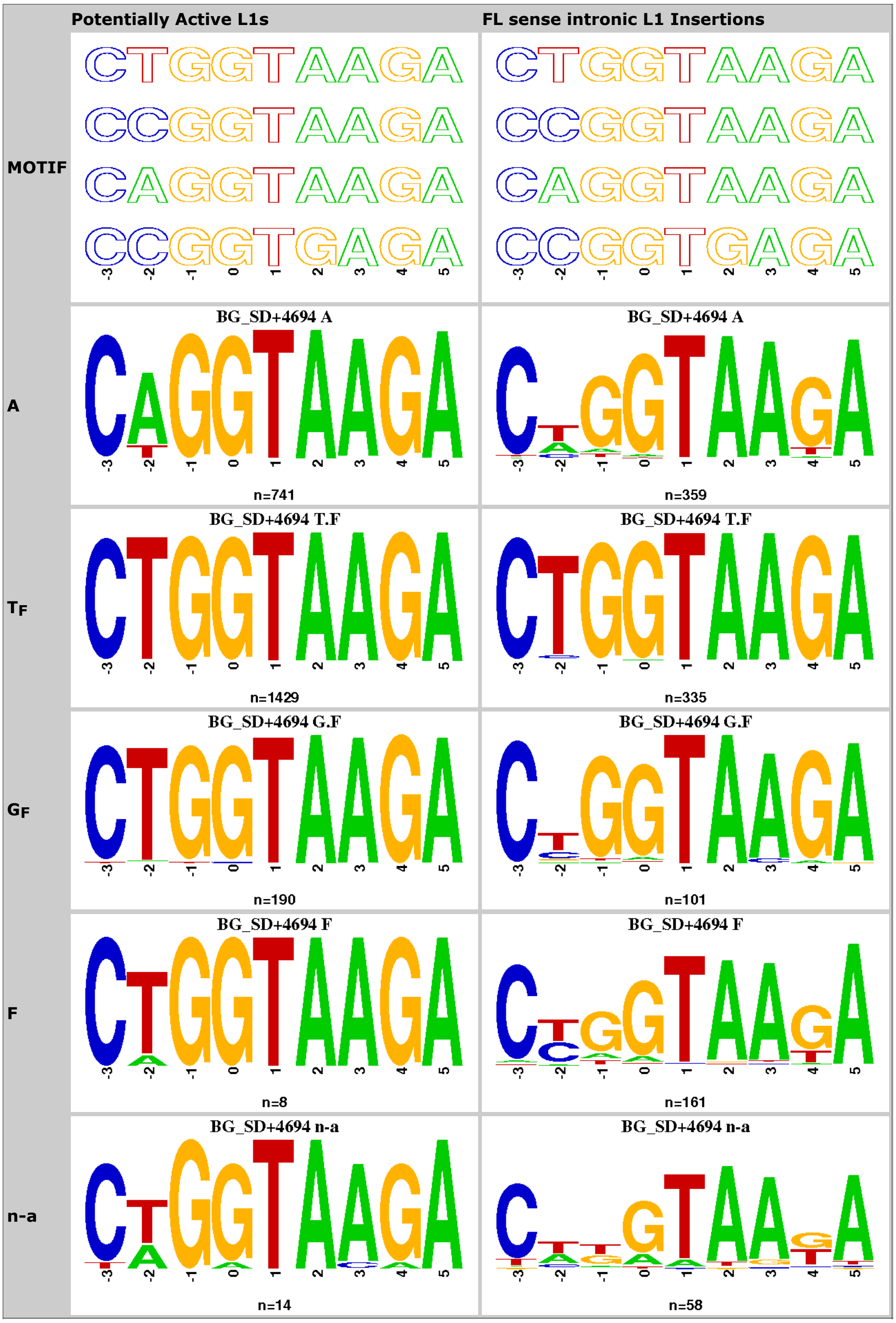
Potentially Active L1s
FL sense intronic L1 Insertions
MOTIF
CTGGTAAGA
CCGGTAAGA
CAGGTAAGA
CCGGTGAGA
-3 -2 -1 0 1 2 3 4 5
A
BG_SD+4694 A
n=741
n=359
TF
BG_SD+4694 T.F
n=1429
n=335
GF
BG_SD+4694 G.F
n=190
n=101
F
BG_SD+4694 F
n=8
n=161
n-a
BG_SD+4694 n-a
n=14
n=58

BG_SD+4903

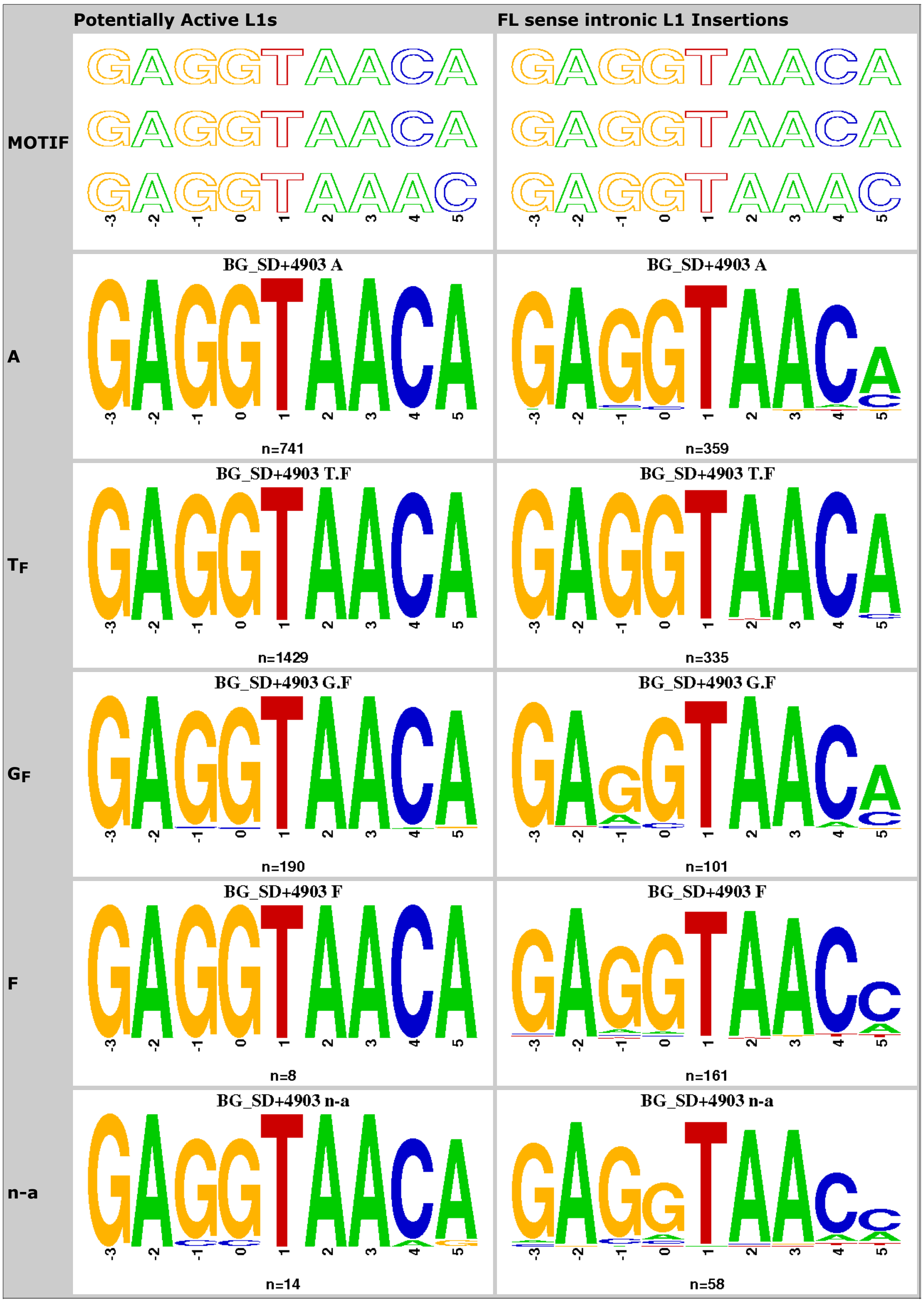

Potentially Active L1s
FL sense intronic L1 Insertions
MOTIF
A
TF
GF
F
n-a
BG_SD+4903 A
n=741
n=359
BG_SD+4903 T.F
n=1429
n=335
BG_SD+4903 G.F
n=190
n=101
BG_SD+4903 F
n=8
n=161
BG_SD+4903 n-a
n=14
n=58
